# Dual targeting of CD244 and CD2 by a viral-immunoevasin-guided engineered CD48-Fc mutant for T- and NK-cell modulation

**DOI:** 10.3389/fimmu.2026.1876221

**Published:** 2026-09-01

**Authors:** Olga Bautista-Cerecero, Pablo Hernández-Luis, Pablo Martínez-Vicente, Francesc Poblador, Narcis Fernandez-Fuentes, Baldomero Oliva, Julen Merino-Aboitiz, Pablo Engel, Ana Angulo

**Affiliations:** 1 Immunology Unit, Department of Biomedical Sciences, Faculty of Medicine and Health Sciences, University of Barcelona, Barcelona, Spain; 2 Josep Carreras Leukemia Research Institute (IJC), Barcelona, Spain; 3 Structural Bioinformatics Laboratory (GRIB-IMIM), Department of Medicine and Life Sciences, Universitat Pompeu Fabra, Barcelona, Spain; 4 Institut d’Investigacions Biomèdiques August Pi i Sunyer, Barcelona, Spain

**Keywords:** CD2, CD244/2B4, CD48 mutein, dual targeting, Fc-fusion protein, immunoevasin-guided design, immunotherapy, T- and NK-modulation

## Abstract

Viruses encode immunoevasins that subvert host co-signaling pathways. CD244:CD48 and CD2:CD58 interactions are critical for NK- and T-cell effector responses. We previously identified a herpesvirus-encoded soluble CD48 homolog that functions as a decoy ligand for CD244 and dampens NK-cell activation. Here, we show that this viral protein, A43, also recognizes CD2, and we used its structural features to engineer human CD48-Fc variants with dual CD244/CD2 specificity. The optimized mutein, C5-Fc, which incorporates twelve A43-guided substitutions predicted by structure-based modeling to enhance interface packing and electrostatic complementarity, resulted in high-avidity binding and competitively displacement of native ligands. Fc-silenced C5-Fc (C5-Fc^#^) disrupted NK- and T-cell conjugate formation and reduced CD244- and CD2-dependent cytotoxicity. It also interfered with the maturation of the antigen-dependent immunological synapse and inhibited T-cell proliferation and the secretion of pro-inflammatory cytokines. These data establish C5-Fc^#^ as a rationally engineered dual-specificity inhibitor with translational potential for autoimmune diseases.

## Introduction

Viruses have evolved sophisticated mechanisms to subvert antiviral immunity through the expression of immunomodulatory proteins (1, 2). Knowledge gained from these potent immunoevasins is key to understand viral pathogenesis and can guide the development of antiviral and immunomodulatory therapeutics. Many viral strategies rely on host genes captured through horizontal gene transfer (3). Over evolutionary time, such genes have diversified to produce molecules that mimic or inhibit innate and adaptive immune pathways, enhancing viral replication (4). Among these, some belong to the immunoglobulin (Ig) superfamily, one of the most diverse protein groups in vertebrates (5).

The signaling lymphocytic activation molecule (SLAM) family, comprising nine Ig superfamily receptors expressed on hematopoietic cells, plays critical roles in cell-cell communication, modulating lymphocyte activation (6, 7). CD48 (SLAMF2) is broadly expressed on leukocytes (8, 9) and serves as a high-affinity ligand for CD244 (2B4/SLAMF4) (10). Both CD48 and CD244 contain extracellular regions composed of an N-terminal, distal Ig variable (IgV) ligand binding domain followed by a membrane-proximal Ig constant 2-set (IgC2) domain. CD48 is a glycosyl-phosphatidylinositol (GPI)-anchored protein, whereas CD244 is a type I transmembrane signaling receptor that contains immune receptor tyrosine-based switch motifs (ITSMs) in its cytoplasmic tail (11, 12). Engagement by CD48:CD244 recruits specific adaptor proteins, including SAP (SH2D1A) and EAT-2 (SH2D1B), to these ITSMs, thereby activating NK and cytotoxic T cells (13). In rodents, CD48 also binds the co-stimulatory molecule CD2 (LFA-2), which is predominantly expressed on T and NK lymphocytes (14, 15). In humans, however, the physiological ligand of CD2 is CD58 (LFA3) (16).

Given its central role in lymphocyte effector function, the CD48:CD244 axis has emerged as a target of viral immune-evasion strategies. We previously reported that mouse cytomegalovirus (CMV) downregulates CD48 through the viral mucin-like protein m154 (17). This protein promotes lysosomal degradation of CD48 by interfering with the adaptor protein-1 (AP-1) complex, impairing NK-cell responses and enhancing viral replication *in vivo* (18). HIV-1 similarly decreases CD48 surface expression on infected CD4^+^ T cells (19, 20), an effect mediated by Vpu to facilitate escape from antibody-dependent cellular cytotoxicity (ADCC) (21).

Viral CD48 homologs (vCD48s) have been identified in several large DNA viruses (5, 22). A notable example is A43, a primate CMV-encoded CD48 mimic functioning as a soluble CD48 decoy (23). A43 has evolved unique structural features, such as the replacement of the GPI anchor with a transmembrane region and cytoplasmic tail. Importantly, the viral protein binds human CD244 with a faster association rate and markedly slower dissociation, resulting in an affinity substantially higher than that of human CD48. Soluble A43 blocks CD48:CD244 interaction, disrupting NK-cell immune synapse formation and reducing cytotoxicity and IFNγ production (23).

Here, we show that A43 also binds human CD2, underscoring its dual receptor specificity. To generate safe, human-derived immunomodulators with minimal immunogenic risk, we engineered soluble human CD48 variants that incorporate key A43-like interaction features. Our lead candidate, C5-Fc^#^, recognizes both human CD244 and CD2 with high affinity, competitively disrupting CD48:CD244 and CD58:CD2 interactions. Functionally, C5-Fc^#^ blunts NK- and T-cell functions, demonstrating the feasibility of designing biotherapeutics inspired by viral immunoevasins to selectively target these two co-stimulatory pathways in autoimmune and inflammatory diseases, as well as in transplant rejection.

## Results

### A43 binds human CD244 and CD2

**Figure 1:**
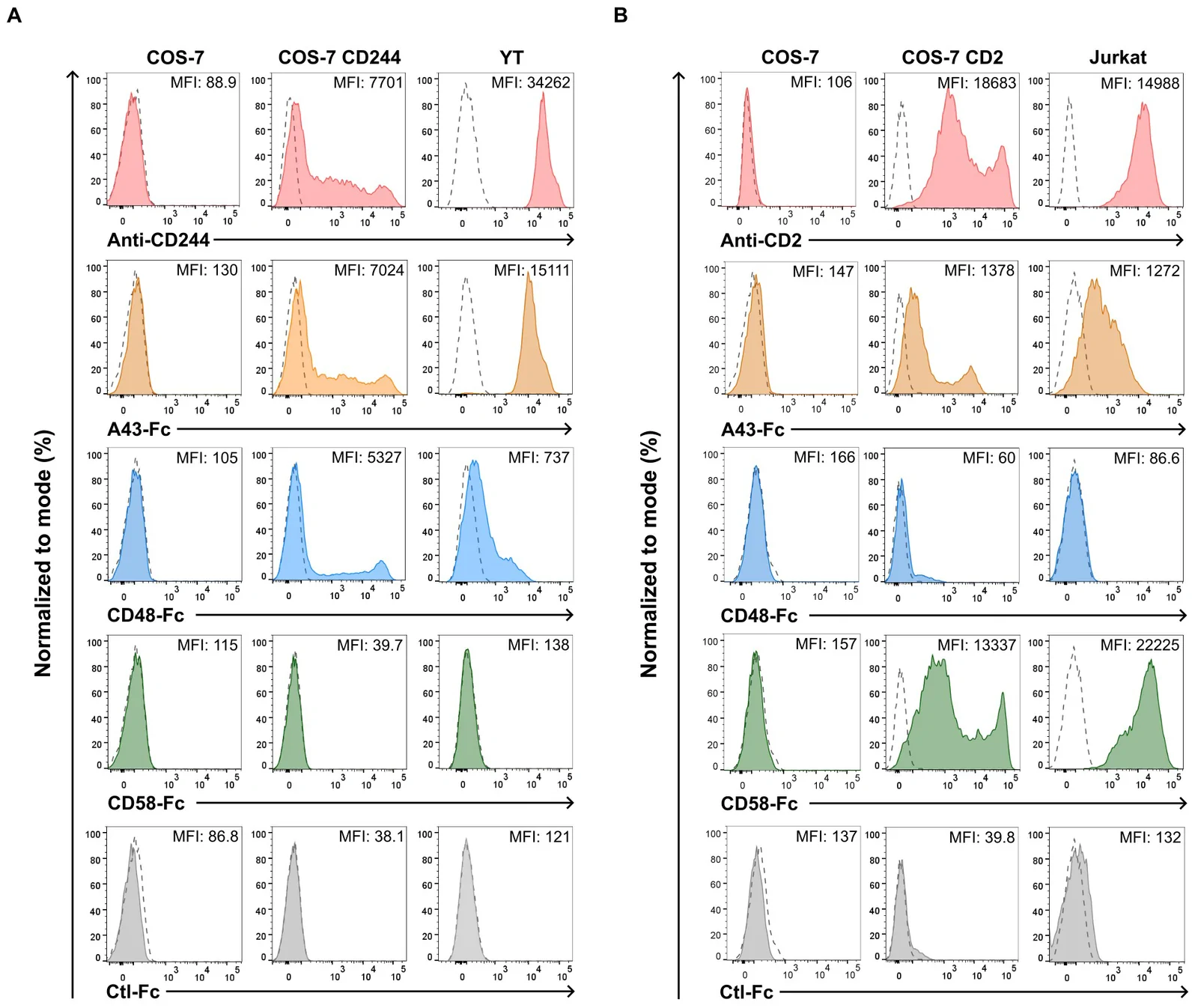
Interaction of A43-Fc with human CD244- and CD2-expressing cells. **(A)** COS-7 cells (non-transfected controls), COS-7 cells transfected with CD244 (COS-7 CD244), and YT cells were analyzed by flow cytometry for CD244 expression (anti-CD244) and binding to 5 μg/mL of A43-Fc, CD48-Fc, CD58-Fc, or an unrelated Fc protein (Ctl-Fc), and detected with an anti-human IgG Fc-PE antibody. Representative histograms and mean fluorescence intensity (MFI) values from duplicate samples out of two independent experiments are shown. Dashed-line histograms refer to cells incubated with anti-human IgG Fc-PE antibody only. **(B)** As in **(A)**, except that COS-7 cells (non-transfected controls), transfected with CD2 (COS-7 CD2), and Jurkat cells were used and CD2 expression (anti-CD2) instead of CD244 was analyzed.

We previously reported that the soluble viral CD48 homolog A43 efficiently binds human CD244 (23). As shown in **Figure 1A**, A43-Fc, consisting of the viral ectodomain of the viral protein fused to the human IgG1 Fc (designated as Fc), bound COS-7 cells transiently expressing human CD244 (COS-7 CD244 cells), but not untransfected COS-7 cells. The viral protein also interacted with YT cells, a human NK-cell line expressing endogenous CD244. Notably, A43-Fc binding to both COS-7 CD244 and YT cells exceeded that of human CD48-Fc, aligning with our previous findings showing that A43 displays an approximately six-fold higher affinity for human CD244 than human CD48 (23). In contrast, no binding was detected with an unrelated Fc-fusion protein (Ctl-Fc), used as a negative control. Because human CD48 is a low-affinity ligand for human CD2 (14), whereas in rodents CD48 serves as the natural ligand for CD2 (24), we examined whether A43 could also recognize human CD2 (**Figure 1B**). Indeed, A43-Fc bound to COS-7 cells transiently transfected with human CD2 (COS-7 CD2 cells). A43-Fc also bound to Jurkat T cells, which endogenously express CD2, although at lower levels than human CD58-Fc protein, the native human CD2 ligand (25). Neither CD48-Fc nor Ctl-Fc showed detectable binding to COS-7 CD2 or Jurkat cells.

While human CD48 does not interact with additional SLAM family members, we explored whether the viral protein displayed broader SLAM family recognition. Flow cytometry analysis revealed no binding of A43-Fc to CD150 (SLAMF1), CD48 (SLAMF2), CD229 (SLAMF3), CD84 (SLAMF5), CD352 (SLAMF6), or CD319 (SLAMF7) expressed on transiently transfected COS-7 cells (**Supplementary Figure 1**). Collectively, these results indicate that the viral A43 protein specifically interacts with human CD244, more strongly than human CD48, and also recognizes human CD2.

### A43-guided design and generation of human CD48-Fc mutants

**Figure 2:**
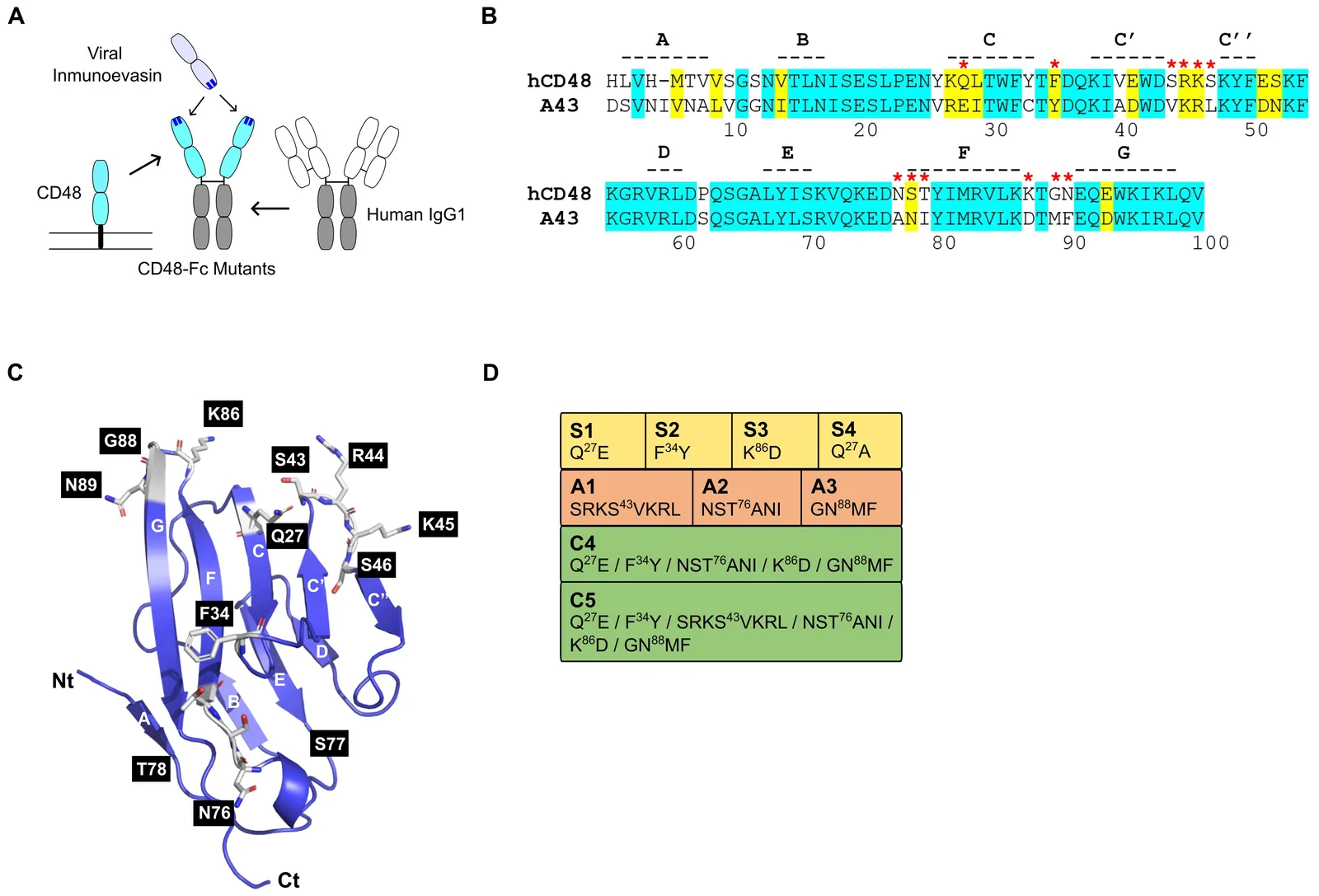
Design and construction of CD48-Fc mutants guided by A43. **(A)** Schematic illustration of the design of a representative human CD48-Fc mutant. **(B)** Alignment of the N-terminal Ig-like domain of human CD48 and A43, with predicted β-strands depicted with dashed lines and labeled using the standardized nomenclature for Ig-like domains. CD48 residues conserved in A43 are shaded in blue (identical amino acid) or yellow (similar amino acid). Red asterisks mark CD48 residues substituted with the corresponding residues from A43. **(C)** Ribbon model of the N-terminal Ig-like domain of human CD48 obtained from the coordinates deposited in the PDB databank (PDB2edo), showing mutated residues in black. β-strands are indicated as arrows. **(D)** Design of a panel of CD48-Fc mutants: single mutants (S1, S2, S3, and S4) in yellow boxes, adjacent mutants (A1, A2, and A3) in orange boxes, and combined mutants (C4, C5) in green boxes. In C mutants, the set of changes are separated by a slash symbol (/). In A and C mutants (A1, A2, and A3), only the position of the first residue of each replaced segment is indicated.

Given its soluble nature and ligand-binding properties, A43 may function as a decoy receptor capable of simultaneously modulating CD2- and CD244-mediated T- and NK-cell responses. This makes it an attractive template for designing biomolecules to dampen excessive immune activation, such as in autoimmune conditions. To generate a safe, dual inhibitor suitable for human application, we rationally engineered mutated versions of human CD48 based on the structural and functional properties from A43. We produced a panel of soluble, A43-guided human CD48 mutants by substituting selected human CD48 residues with the corresponding viral amino acids, with the aim of enhancing and broadening receptor-blocking capacity. All mutants were expressed as bivalent human IgG1 Fc-fusion proteins (-Fc) comprising both extracellular Ig-like domains of human CD48, with the second domain left intact (**Figure 2A**). Together with these molecules, human CD48-Fc, human CD58-Fc, and an unrelated Fc-fusion protein were employed as controls.

To identify key residues underlying the distinct interaction profiles of A43 and human CD48, we aligned their N-terminal Ig-like domains, which display 66% sequence identity and 84% similarity, focusing on the CC′C″ face and FG loop implicated in receptor engagement (**Figure 2B**) (26, 27). Mutations were introduced at specific positions differing between CD48 and A43, including residues known to contact CD244 and CD2 (26–28). Residues exchanged by the corresponding ones in A43 were Q^27^, F^34^, K^86^, G^88^ and N^89^ (marked with red asterisks in **Figure 2B**). Additionally, we mutated the NST glycosylation site (residues 76-78) preceding β-strand F, and the SRKS sequence (residues 43-46) in the C’C’’ loop, which we hypothesized could influence folding, stability, or ligand accessibility. A structural model of CD48 highlighting these residues is shown in **Figure 2C**. We generated several mutant classes incorporating A43 substitutions: The S series (S1, S2, and S3) with single-residue changes; the A series (A1, A2, and A3) bearing short stretches of 2–4 amino acids replacements; and the C series (C4, and C5) combining both types of modifications (**Figure 2D**). Importantly, even the introduction of all 12 mutations in the CD48-Fc mutant C5 did not cause major structural perturbations (**Supplementary Figure 2**). Rather, these mutations produced only localized changes confined to the immediate environment of the substituted residues while preserving the overall structural architecture of CD48. An alanine-substituted control mutant, S4, was also included.

### Engineered CD48-Fc mutants display improved binding capacities to both human CD244 and CD2

**Figure 3:**
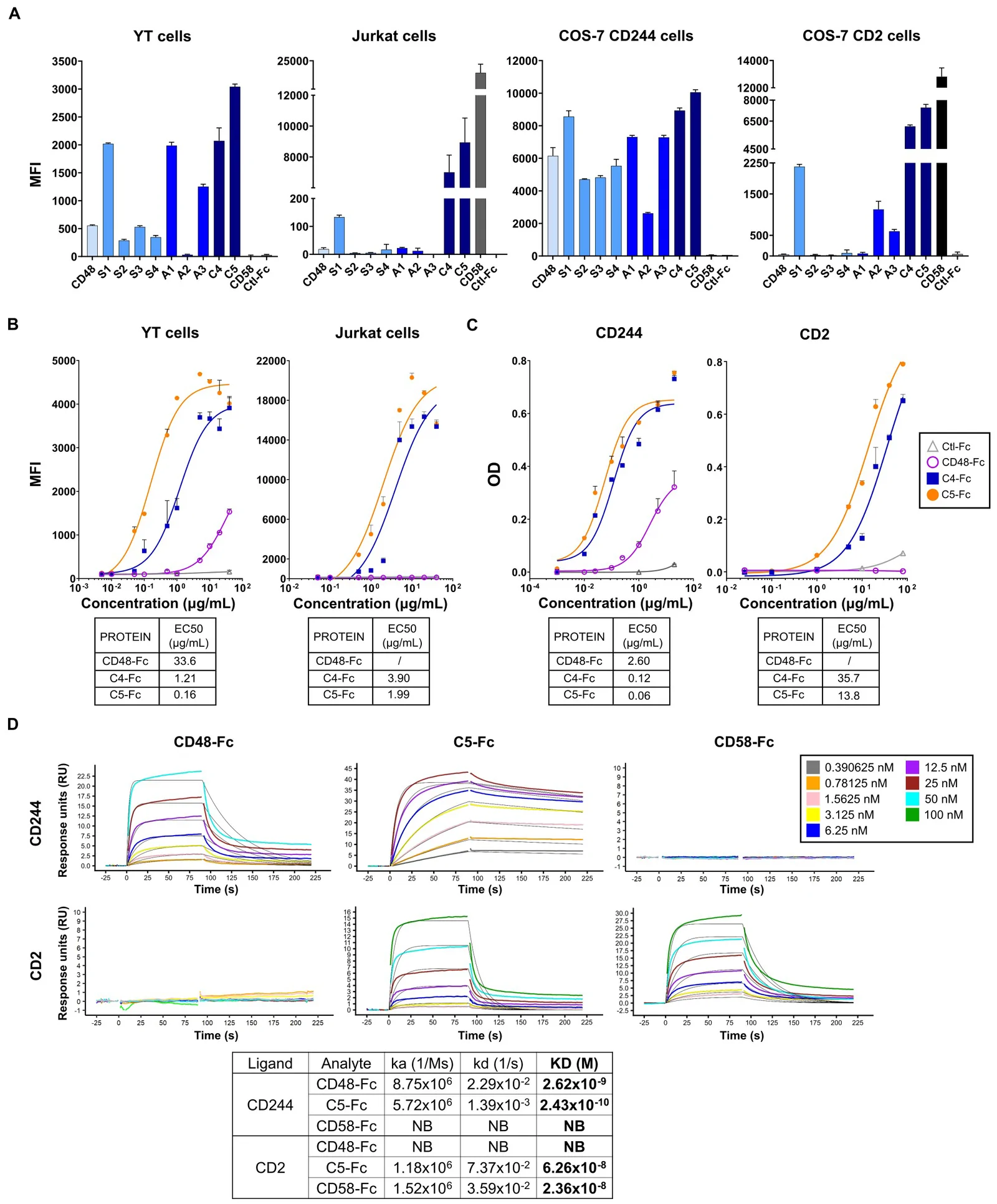
Binding properties of CD48-Fc mutants with CD244 and CD2. **(A)** YT cells, Jurkat cells, or COS-7 cells expressing CD244 or CD2 were incubated with CD48-Fc, S1-Fc, S2-Fc, S3-Fc, S4-Fc, A1-Fc, A2-Fc, A3-Fc, C4-Fc, C5-Fc, CD58-Fc, or Ctl-Fc. Fc proteins were used at 5 μg/mL (YT, Jurkat, and COS-7 CD2 cells) or 0.25 μg/mL (COS-7 CD244 cells). Binding was detected using anti-human IgG Fc-PE antibody and analyzed by flow cytometry. MFI values are shown as mean ± standard deviations (SD) of duplicate samples from one out of two representative experiments. **(B)** YT and Jurkat cells were incubated with increasing concentrations of purified CD48-Fc, C4-Fc, C5-Fc, or Ctl-Fc and analyzed by flow cytometry after staining with anti-human IgG Fc-PE. Curves represent MFI values across various concentrations of Fc-fusion proteins. Data are mean ± SD of duplicate samples from one of two representative experiments. Values of the half-maximal effective concentration (EC50) are indicated. **(C)** Immobilized His-tagged CD244 or CD2 proteins (2 μg/mL) were incubated with increasing concentrations of purified CD48-Fc, C4-Fc, C5-Fc, or Ctl-Fc. Binding was quantified by ELISA using an anti-human IgG Fc-POD (OD 450–470 nm). Curves show mean ± SD of duplicate samples from a representative experiment out of two. EC50 values are indicated. **(D)** His-tagged human CD244 and CD2 were immobilized on CM5 sensor chips via anti-histidine antibodies. Serial dilutions of purified CD48-Fc, C5-Fc, and CD58-Fc proteins were injected at 30 μL/min. Sensorgrams and fittings curves are shown for CD48-Fc: CD244, C5-Fc: CD244, CD58-Fc: CD244, CD48-Fc: CD2, C5-Fc: CD2, and CD58-Fc: CD2 interactions measured by SPR analysis. Kinetic and affinity constants appear in the accompanying table. NB denotes no binding.

We first assessed by flow cytometry CD48-Fc mutant binding to endogenous CD244 and CD2 using YT and Jurkat cell lines, respectively (**Figure 3A**, left two panels). Among single-residue mutants, only S1-Fc (Q^27^E) presented a substantial increase in binding to both CD244 and CD2, whereas the mutant S4-Fc (Q^27^A) showed no improvement over CD48-Fc, underscoring the critical role of Q^27^ for the dual ligand recognition. When evaluating mutants comprising short adjacent residue stretches, A1-Fc (SRKS^43^VKRL) displayed a markedly enhanced CD244 binding, but not CD2 binding. The relevance of these C’C’’ loop residues was further confirmed by the differential CD244 interaction between C4-Fc and C5-Fc, which differ only by the inclusion of these four residues. Mutations in A3-Fc (GN88MF) also improved CD244 binding, without significantly affecting CD2 interaction. Notably, the strongest dual-binding phenotypes were observed for the combined mutants C4-Fc (Q^27^E/F^34^Y/NST^76^ANI/K^86^D/GN^88^MF) and C5-Fc (Q^27^E/F^34^Y/SRKS^43^VKRL/NST^76^ANI/K^86^D/GN^88^MF), containing 8 and 12 substitutions respectively. Both variants exhibited markedly elevated binding to CD244 and acquired robust binding to CD2, with C5-Fc showing the best interactions, overall. While CD58-Fc did not recognize CD244, it exhibited a stronger binding to its native partner CD2, than any of the CD48 mutants. As anticipated, the unrelated Ctl-Fc protein showed no significant binding to either ligand. Mutant binding was next evaluated using COS-7 cells transiently expressing CD244 or CD2 (**Figure 3A**, right two panels). Binding patterns mirrored those observed with YT and Jurkat cells, although in this case A2-Fc (NST^76^ANI) and A3-Fc (GN^88^MF) also showed some binding to CD2.

### C5-Fc exhibits enhanced dual binding and increased reactivity with CD244 and CD2

We selected CD48-Fc mutants C4-Fc and C5-Fc, which displayed the strongest interactions with CD244 and CD2, for detailed dose-response analyses using the two receptors expressed on the cell surface or in solution. Flow cytometry assays using YT cells showed substantially enhanced binding of both mutants relative to CD48-Fc, with C5-Fc exhibiting the greatest increase across all concentrations tested (**Figure 3B**, left panel). Binding was dose dependent, whereas the unrelated Fc protein showed no interaction. Similar results were obtained with Jurkat cells, although in this case, CD48-Fc displayed negligible binding even at the highest concentration tested (**Figure 3B**, right panel). EC50 values for C5-Fc were approximately 7.6-fold and 2-fold lower than those of C4-Fc in YT and Jurkat cells, respectively. ELISA assays employing immobilized CD244 or CD2 His-tagged proteins and different concentrations of the Fc-fusion proteins confirmed these findings (**Figure 3C**). In these assays, EC50 values for C5-Fc were 2-fold (CD244) and 2.6-fold (CD2) lower than those of C4-Fc. Given its consistently superior binding to CD244 and CD2 in both cellular and solution-based assays, and therefore the greatest potential to inhibit CD48:CD244 and CD58:CD2 interactions, C5-Fc was selected for further characterization.

To complement these results, surface plasmon resonance (SPR) kinetic analyses were additionally conducted to quantify real-time interactions between C5-Fc and soluble CD244 and CD2, using CD48-Fc and CD58-Fc as controls. Histidine-tagged human CD244 and CD2 were captured on CM5 sensors, and the binding of serial dilutions of purified Fc-fusion proteins was evaluated (**Figure 3D**). C5-Fc interacted with CD244 with an association rate (Ka) of 5.72 x 10^6^ M^-1^ s^-1^ and a dissociation rate (Kd) of 1.39 x 10^−3^ s^-1^, yielding an equilibrium dissociation constant (KD) of 0.2 nM. Notably, compared with CD48-Fc, C5-Fc demonstrated a 10.8-fold higher affinity and a 16.5-times slower dissociation. As anticipated, binding of CD58-Fc to CD244 was not observed. For CD2, C5-Fc reached a KD of 62 nM, approaching that of CD58-Fc (23 nM). In agreement with previous reports using this technique, no CD48-Fc interaction with CD2 was detected (29). Overall, these findings demonstrate a markedly improved affinity and kinetics of the C5-Fc molecule.

Importantly, the specificity of C5-Fc for CD244 and CD2 was supported by its lack of interaction with other SLAM family receptors expressed on the surface of COS-7 cells (**Supplementary Figure 1**).

### Structural modeling of C5:CD244 and C5:CD2 complexes

**Figure 4:**
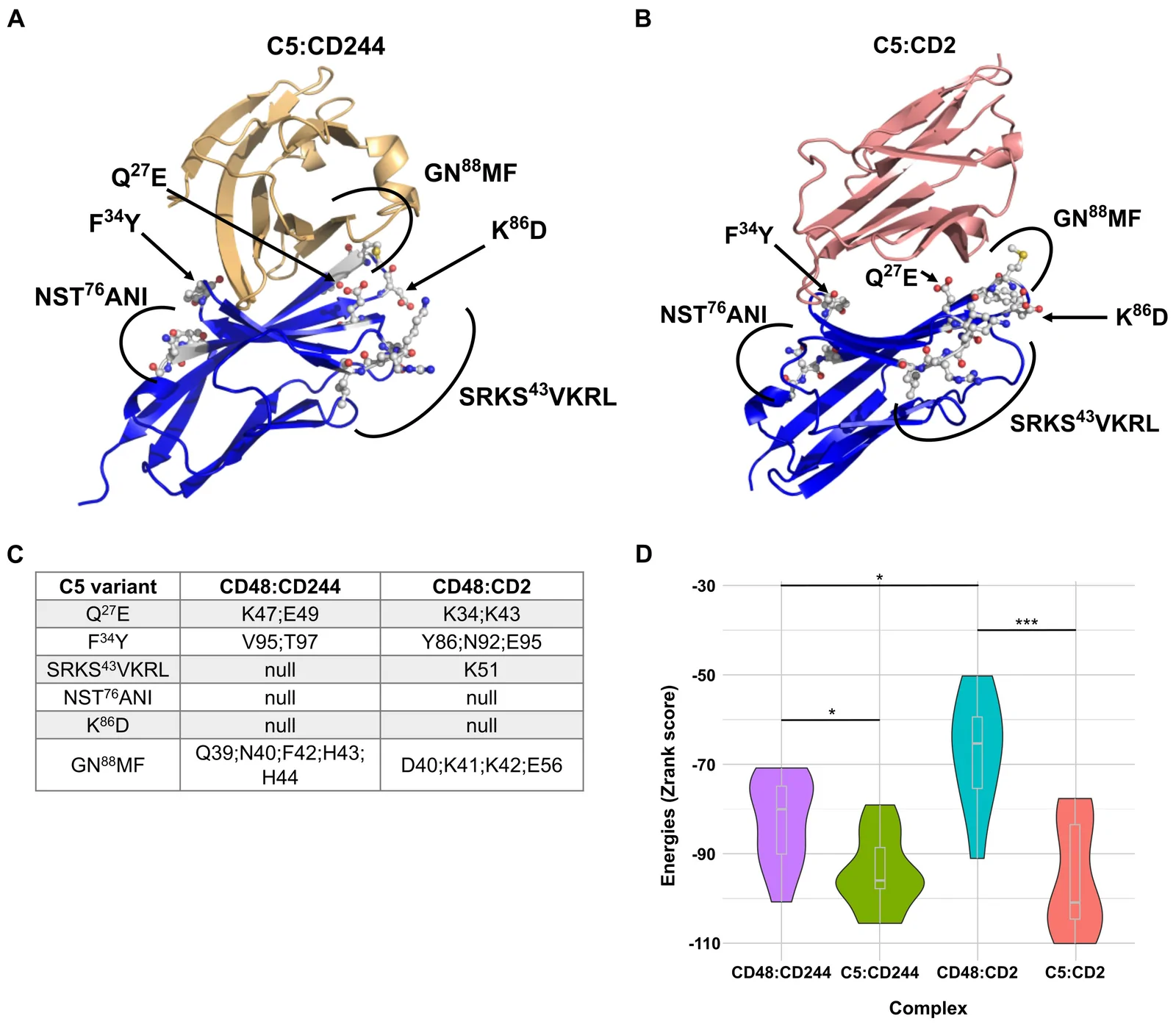
Structural model of the C5 variant in complex with CD244 and CD2. **(A, B)** Ribbon model of the C5 variant (blue) bound to CD244 (light brown) and CD2 (salmon). Engineered mutations in C5-Fc are labeled and shown in ball-and-stick format, with C, N, and O atoms in white, blue, and red, respectively. **(C)** Table summarizing atomic interactions between C5-Fc mutated residues and CD244 and CD2; ‘null’ denotes no interaction. **(D)** Violin plots showing binding-energy distributions for the CD48:CD244, C5:CD244, CD48:CD2, and C5:CD2 models, calculated with ZRANK. Statistically significant differences between distributions are indicated by asterisks. *p<0.05, ***p<0.001.

To gain further understanding of the molecular basis of C5 recognition of CD244 and CD2, we generated structural models of both complexes, as the ultrastructures of the CD48:CD244 and CD58:CD2 complexes have so far only been solved in rodents. The overall architecture of the C5 mutant bound to CD244 (**Figure 4A**) and CD2 (**Figure 4B**) is highly similar, with each interaction dominated by β-strands in the central “core” region and supported by peripheral loop contacts (“rim”). Aside from Q^27^E, located in strand C, at the core of the interface, the remaining substitutions map to rim regions (see also **Figures 2B, C**). In terms of interface area, C5:CD244 and C5:CD2 complexes are comparable, measuring 1554 Ang^2^ and 1602 Ang^2^, respectively.

The models clarify how individual C5 substitutions contribute to ligand binding (**Figure 4C**). Among single-residue replacements, Q^27^E and F^34^Y primarily contribute to non-bonded polar interactions with 2–3 residues in either partner, whereas K^86^D lies outside of the interface and does not contact CD244 or CD2. Of the adjacent-residue substitutions, GN^88^MF (positions 88-89) forms extensive contacts with both partners, engaging 5 residues in CD244 and 4 in CD2. The SRKS^43^VKLR change in the C’C’’ loop (residues 43-46) participates in the interaction with CD2 residue K^51^, but not with CD244, due to a greater loop-loop separation in the CD244 complex (**Figures 4A, B**). Likewise, NST^76^ANI (residues 76-78), which includes a glycosylation site preceding β-strand F, is positioned away from the interfaces and contributes no detectable contacts.

To quantify the affinity of these atomic interactions, we predicted binding energies for each complex and compared them with those of CD48. As shown in **Figure 4D**, both C5:CD244 and C5:CD2 complexes exhibited markedly lower binding energies than the corresponding CD48 complexes, indicating a substantially increased interaction. These predictions aligned with our flow cytometry, ELISA and SPR binding data, together demonstrating that the engineered substitutions in C5-Fc significantly enhanced its affinity for both CD244 and CD2.

### Binding competition of CD48 to CD244 and CD58 to CD2 by C5-Fc

**Figure 5:**
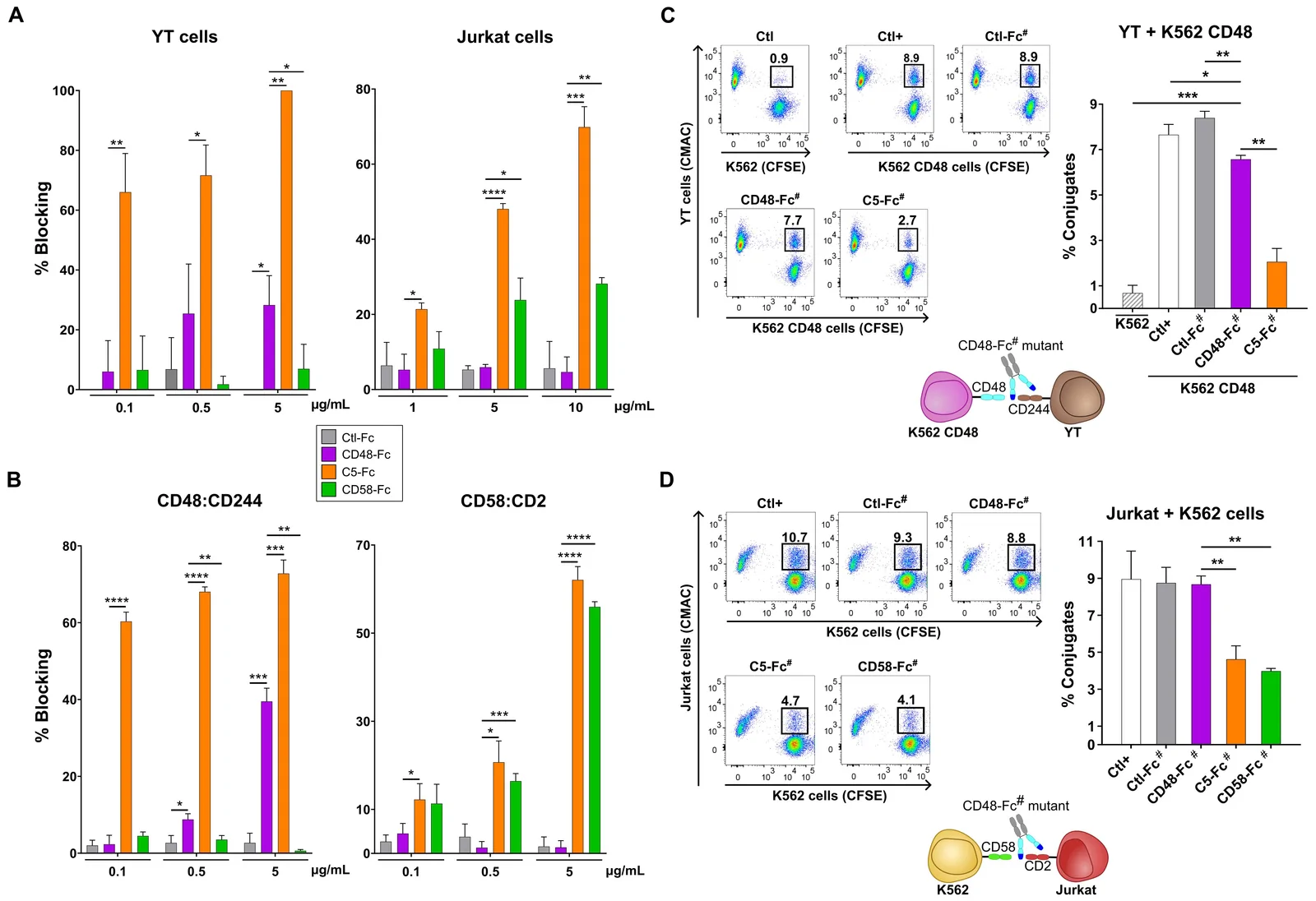
Blockade of the formation of CD48:CD244 and CD58:CD2 conjugates by C5-Fc^#^. **(A)** YT cells and Jurkat cells were incubated with the indicated concentrations of purified CD48-Fc, C5-Fc, CD58-Fc, or Ctl-Fc, followed by CD48-mFc (for YT cells) or CD58-mFc (for Jurkat cells) and analyzed by flow cytometry using anti-mouse IgG Fc-PE. The percentage of interaction blockage was calculated from MFI values. Data represent mean ± SD of triplicate samples from a representative experiment out of two. **(B)** Plates coated with 3 μg/mL of purified CD244-Fc and CD2-Fc were incubated with the indicated concentrations of purified CD48-Fc, C5-Fc, CD58-Fc or Ctl-Fc, followed by CD48-mFc (CD244-Fc plates) or CD58-mFc (CD2-Fc plates) and analyzed by ELISA using anti-mouse IgG Fc-POD. OD 450–470 nm readings were performed in triplicates and used to calculate the percentage of blockade of CD48:CD244 and CD58:CD2 binding. Data represent mean ± SD of triplicate samples from a representative experiment out of two. **(C)** CFSE-labeled K562 cells untransfected or stably transfected with CD48 (K562 CD48) were incubated at 37°C with CMAC-labeled YT cells at a 1:1 ratio, in the absence (Ctl+) or presence of 5 μg/mL of purified CD48-Fc^#^, C5-Fc^#^ or Ctl-Fc^#^. Conjugates, identified as double positive cells by flow cytometry, were quantified at 0 and 20 min. Representative dot plots are depicted with conjugate percentages indicated. A schematic diagram illustrating the conjugate competition assay is shown. The right panel depicts a graphic representation of the percentage of conjugates formed at 20 min after subtracting conjugates present at 0 min. Data represent the mean ± SD of triplicate samples out of two independent experiments. **(D)** As in **(C)**, except that Jurkat cells were used instead of YT cells, and K562 cells instead of CD48-transfected K562 cells. CD58-Fc^#^ was also included in the assay. Asterisks indicate statistically significant differences relative to the CD48-Fc or CD48-Fc^#^ condition *p<0.05, **p<0.01, ***p<0.001, ****p<0.0001.

We assessed whether C5-Fc can compete with the natural interactions of CD48 and CD58 with CD244 and CD2, respectively. Competition was measured using ligands displayed on the cell surface or presented in solution. For cell-based assays, YT cells and Jurkat cells were incubated with C5-Fc together with CD48-murine Fc (CD48-mFc) or CD58-murine Fc (CD58-mFc), two fusion proteins containing the corresponding ectodomains fused to the mouse IgG1 Fc region. Flow cytometry analysis revealed strong dose-response inhibition of both CD48:CD244 and CD58:CD2 interactions by C5-Fc (**Figure 5A**). Notably, C5-Fc blocked binding far more effectively than CD48-Fc, whereas the unrelated Ctl-Fc protein had no effect. Interestingly, C5-Fc inhibited CD58:CD2 binding even more strongly than CD58-Fc itself.

To validate these findings, we performed competition ELISAs using plates coated with purified CD244-Fc or CD2-Fc, which were incubated with increasing concentrations of C5-Fc, CD48-Fc, CD58-Fc, or Ctl-Fc, followed by CD48-mFc (for CD244 assays), or CD58-mFc (for CD2 assays). Consistent with the cell-based experiments, C5-Fc inhibited CD48:CD244 binding in a clear, concentration dependent manner and substantially more efficiently than CD48-Fc (**Figure 5B**, left panel). Similarly, C5-Fc, interfered with the CD58:CD2 interaction (**Figure 5B**, right panel), whereas CD48-Fc or Ctl-Fc showed no inhibition. The potency of C5-Fc slightly exceeded that of CD58-Fc. Together, these results demonstrate that the engineered C5-Fc mutant effectively outcompetes the natural CD48:CD244 and CD58:CD2 interactions, displaying markedly enhanced blocking capacity relative to CD48-Fc and equivalent or superior potency to CD58-Fc.

### C5-Fc^#^ inhibits conjugate formation between NK cells and CD48-expressing targets and between T cells and CD58-expressing targets

In the next set of experiments, we investigated whether soluble C5-Fc possesses functional activity. For these assays, the Fc region of this protein was modified to include mutations L^234^A and L^235^A (designated as Fc^#^) to reduce antibody-dependent cell-mediated cytotoxicity and complement-dependent cytotoxicity. Parallel Fc^#^ versions of CD48, CD58, and the unrelated control protein were also generated. We first assessed the ability of C5-Fc^#^ to disrupt conjugate formation between NK cells and CD48-expressing targets, as well as between T cells and CD58-expressing cells, interactions which are essential for effective NK- and T-cells effector function. We used a flow cytometry-based assay, in which YT or Jurkat cells were labeled with a cell tracker dye and mixed at a 1:1 ratio, with CFSE-labeled CD48-expressing K562-CD48 or K562 cells, respectively. Conjugates were identified as double-positive events after 20 min co-incubation at 37°C. As shown in **Figure 5C**, YT cell conjugation strictly depended on CD48 expression on K562 cells. Notably, C5-Fc^#^ caused a strong reduction in conjugates formed between YT and K562-CD48 cells, whereas CD48-Fc^#^ produced a weaker inhibition and the unrelated Fc^#^ protein had no effect. Similarly, C5-Fc^#^ substantially blocked conjugate formation between Jurkat cells and CD58^+^ K562 cells, with an inhibitory capacity comparable to CD58-Fc^#^, while the Ctl-Fc^#^ protein had no impact on this process (**Figure 5D**). These data demonstrate that both CD48:CD244-dependent and CD58:CD2-dependent cellular conjugates can be effectively interfered by C5-Fc^#^.

### CD244- and CD2-dependent NK-cell cytotoxicity is impaired by C5-Fc^#^

**Figure 6:**
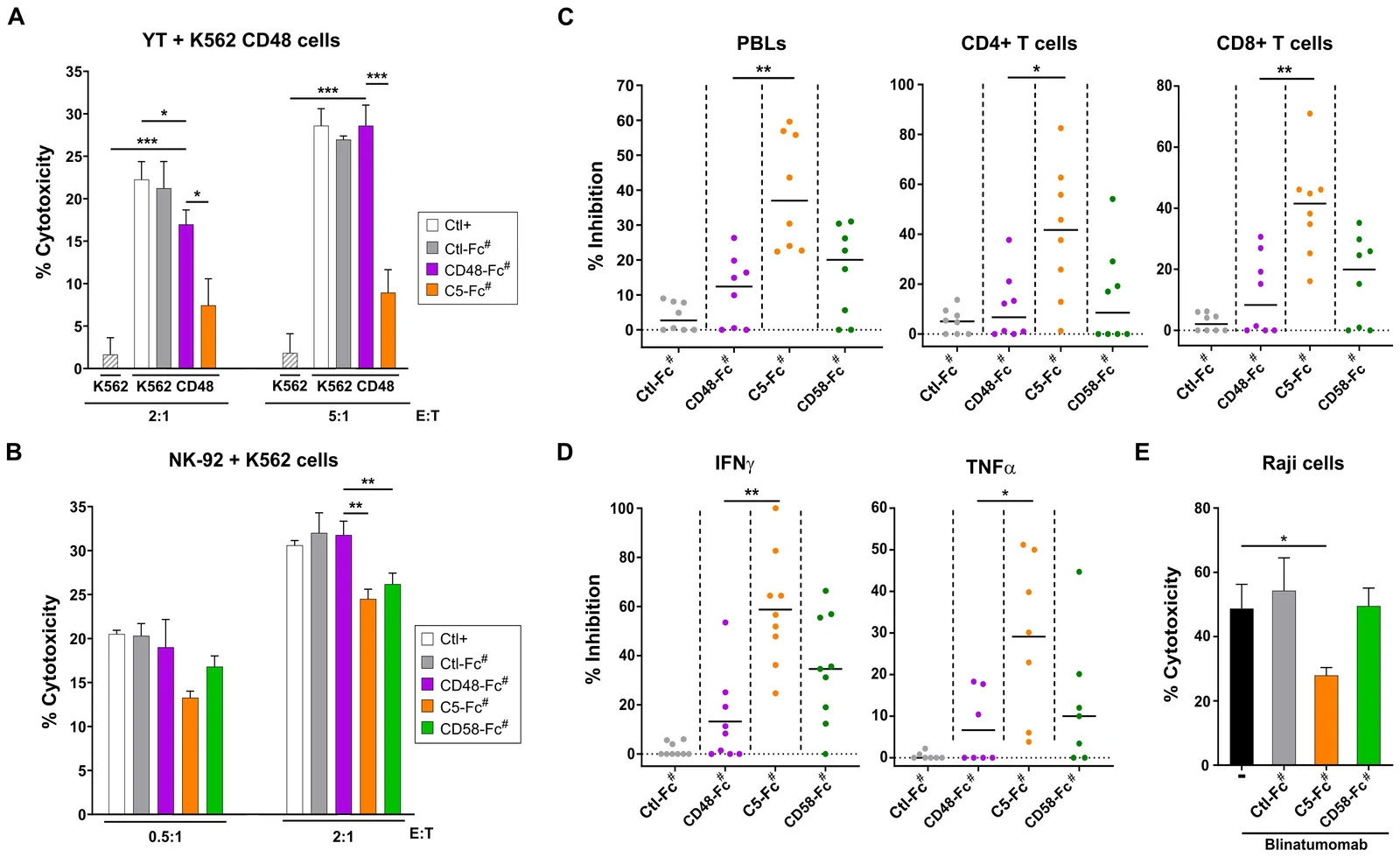
Inhibition of CD244- and CD2-mediated NK-cell cytotoxicity and suppression of T-cell proliferation and cytokine production by C5-Fc^#^. **(A)** YT cells were pre-incubated with 5 μg/mL of purified CD48-Fc^#^, C5-Fc^#^, Ctl-Fc^#^, or left untreated (Ctl+) and subsequently exposed to calcein-labeled CD48-transfected K562 target cells at the E:T ratios indicated for 4 h at 37°C. Calcein-labeled non-transfected K562 target cells were used as controls. Cytotoxicity was quantified by calcein release. Data represent the mean ± SD of triplicate samples from one out of two independent experiments. **(B)** As in **(A)**, except that NK-92 cells instead of YT cells were used in co-cultures with calcein-labeled K562 target cells. CD58-Fc^#^ was additionally included. **(C)** CFSE-labeled responder PBLs were stimulated with irradiated allogeneic PBLs at a 1:1 ratio for 6 days in the presence or absence of 5 μg/mL of purified CD48-Fc^#^, C5-Fc^#^, CD58-Fc^#^, or Ctl-Fc^#^. CFSE-labeled responder PBLs stimulated with irradiated autologous PBLs in the absence of Fc^#^ proteins served as controls. Proliferation of total, CD4^+^, and CD8^+^ T cells was quantified in eight donor pairs by CFSE dilution via flow cytometry. Proliferation measured in the autologous co-culture controls was subtracted. The percentage of inhibition reflects reduced proliferation relative to these controls. Each symbol represents a donor pair; horizontal bars denote mean values. **(D)** Supernatants from nine (IFNγ) or seven (TNFα) donor-pair MLR co-cultures were analyzed on day 6 for IFNγ and TNFα by ELISA. Percentage inhibition corresponds to the reduction in IFNγ or TNFα relative to control co-cultures without Fc^#^ proteins. Each symbol denotes a donor pair; horizontal bars indicate mean values. Asterisks in A, B, C, and D mark statistically significant differences relative to CD48-Fc^#^ exposed samples. **(E)** CellTrace Far Red-labeled Raji target cells were co-cultured with PBMC effector cells at an E:T ratio of 10:1. Cultures were incubated for 24 h in the presence of 0.1 ng/mL blinatumomab biosimilar (CD3×CD19 bispecific antibody) and 5 µg/mL of the indicated purified Fc^#^-fusion proteins. Cytotoxicity was quantified by flow cytometry analysis as the reduction in the number of CellTrace-positive Raji cells. Data represent the mean ± SD of triplicate samples from one out of two independent experiments. Asterisks denote statistically significant differences relative to samples unexposed to Fc^#^-fusion proteins. *p<0.05, **p<0.01,***p<0.001.

We asked whether C5-Fc^#^ modulates NK-cell cytotoxic responses mediated through CD244 or CD2. We first focused on the CD48:CD244 interaction. Cytotoxicity assays were performed using YT cells as effectors and calcein-labeled K562 or CD48-expressing K562 as targets. As expected, YT cells killed CD48-expressing K562 cells far more efficiently than parental K562, confirming CD244-dependent enhancement of cytotoxicity (**Figure 6A**). Importantly, C5-Fc^#^ markedly reduced the killing of CD48^+^ targets at both E:T ratios tested and was substantially more inhibitory than CD48-Fc^#^, whereas Ctl-Fc^#^ showed no effect. We next examined the contribution of the CD58:CD2 interaction to NK-cell cytotoxicity, using NK-92 cells as effectors and K562 cells, which endogenously express CD58, as targets. Despite the relatively low expression of CD2 on NK-92 cells, C5-Fc^#^ consistently produced a modest inhibition of NK-92-mediated lysis at both E:T ratios. This inhibitory effect was slightly more pronounced than that observed with CD58-Fc^#^ (**Figure 6B**). The control Fc^#^ protein did not altered cytotoxicity. Overall, these results show that C5-Fc^#^ significantly diminishes both CD244- and CD2-dependent NK-cell cytotoxicity, consistent with its strong blockade of the corresponding receptor-ligand interactions.

We next sought to determine whether C5-Fc^#^ modulates other NK-celll effector functions by evaluating antibody-dependent cellular cytotoxicity (ADCC) mediated by NK cells using an anti-CD52 monoclonal antibody. As shown in **Supplementary Figure 3**, C5-Fc^#^ produced modest reductions in ADCC against CD3^+^, CD4^+^, and CD8^+^ PBMC subsets, although the effect reached statistical significance only in the CD8^+^ population. These results indicate that C5-Fc^#^ has a limited effect on CD16-mediated ADCC under the conditions tested, although a measurable inhibitory effect was observed in specific target cell populations.

### C5-Fc^#^ reduces proliferation and cytokine production in a mixed lymphocyte reaction assay

To evaluate the impact of C5-Fc^#^ on T-cell responses, we tested its effects on T-cell proliferation and cytokine production in one-way mixed lymphocyte reactions (MLRs). CFSE-stained responder peripheral blood lymphocytes (PBLs) were co-cultured with irradiated allogeneic stimulator PBLs at a ratio of 1:1 for 6 d in the presence of C5-Fc^#^, CD48-Fc^#^, CD58-Fc^#^ or Ctl-Fc^#^. First, the proliferation of responder T cells was measured by flow cytometry, considering the reduction in CFSE intensity as an indicator, in a set of donor pairs. As shown in **Figure 6C** (left panel), responder proliferation varied across donor pairs but was consistently diminished by C5-Fc^#^. Both CD4^+^ and CD8^+^ T cell subsets showed a similar decrease (**Figure 6C**, middle and right panels). Notably, inhibition of C5-Fc^#^ was higher than that of CD58-Fc^#^. We next determined the levels of two key cytokines, IFNγ and TNFα, in MLR culture supernatants. C5-Fc^#^ markedly decreased secretion of both cytokines across all donors, with particularly strong reduction of IFNγ (**Figure 6D**). Conversely, the amount of IFNγ and TNFα released was not significantly affected by Ctl-Fc^#^. Hence, C5-Fc^#^ attenuates T-cell proliferation and dampens pro-inflammatory cytokine production in MLR assays.

### Effect of C5-Fc^#^ on T cell-mediated cytotoxicity

To investigate whether C5-Fc^#^ modulates T cell-mediated cytotoxic responses, we employed a redirected cytotoxicity assay. Because antigen-specific cytotoxic T cell clones were not available, CD19-expressing Raji cells were used as target cells and PBMCs as effector cells in the presence of Blinatumomab, a CD19×CD3 bispecific antibody. This system enables the recruitment and activation of endogenous T cells independently of their native TCR specificity and provides a robust model for assessing T cell-dependent cytotoxicity. Under these conditions, PBMCs efficiently induced lysis of Raji target cells. The addition of C5-Fc^#^ significantly reduced target cell killing compared with the condition lacking Fc^#^-fusion protein, whereas Ctl-Fc^#^ protein had no effect on target cell killing (**Figure 6E**). These findings demonstrate that C5-Fc^#^ is capable of attenuating T cell-mediated cytotoxic activity and extend its inhibitory effects beyond lymphocyte activation responses to include effector functions involved in target cell elimination.

### C5-Fc^#^ disrupts the formation of the antigen-dependent immunological synapse

**Figure 7:**
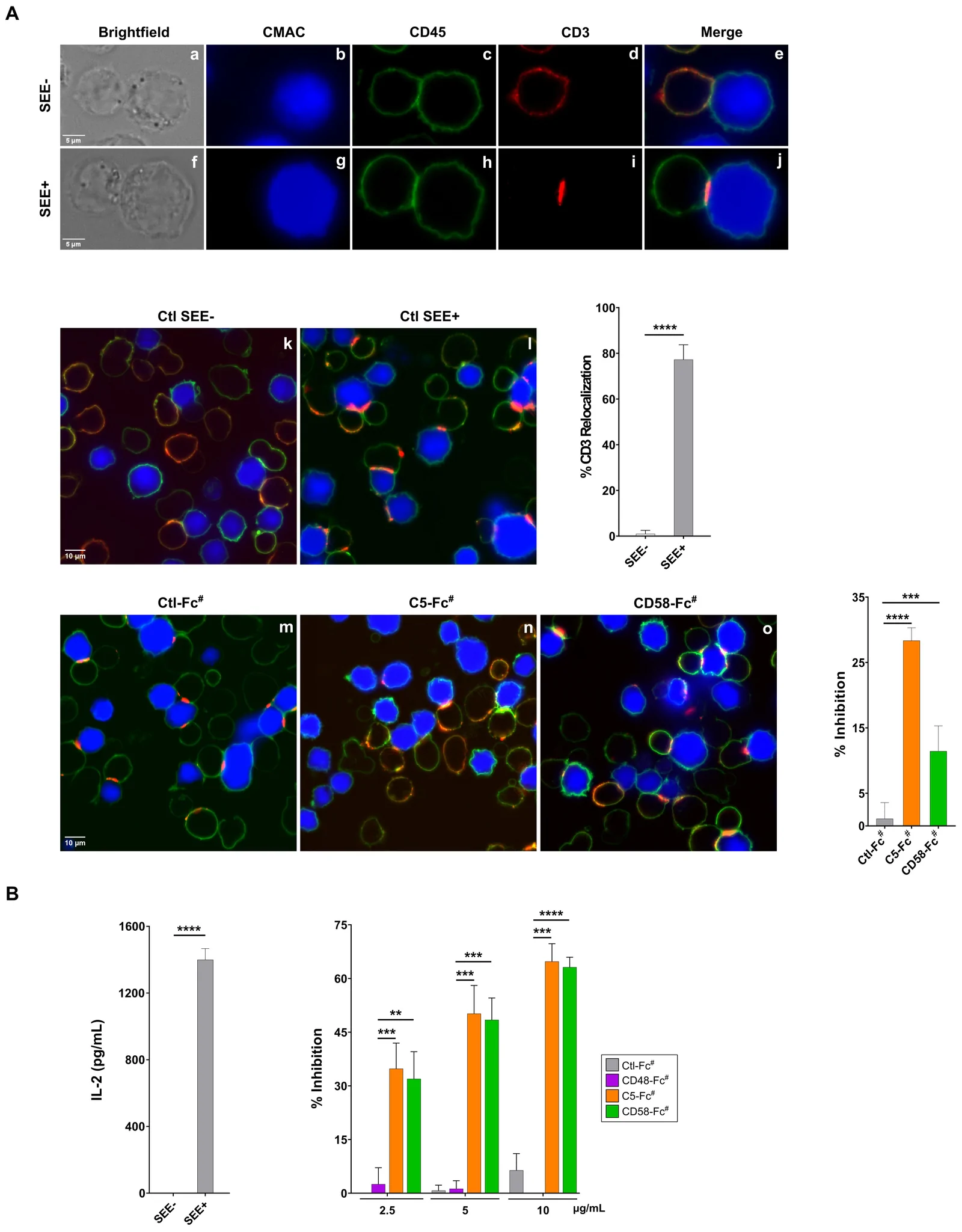
C5-Fc^#^ limits the establishment of the T-cell immunological synapse. **(A)** Jurkat cells were pre-treated with 5 μg/mL of purified C5-Fc^#^, CD58-Fc^#^, Ctl-Fc^#^, or left untreated, and incubated with unpulsed (SEE-) or SEE-pulsed (SEE+) CMAC-labeled Raji cells at a 1:1 ratio for 20 min to allow conjugate formation. Cells were seeded onto poly-L-lysine-coated plates, fixed and stained with anti-CD3 and anti-CD45 antibodies, and analyzed by fluorescence microscopy. Representative stainings of conjugates formed with unpulsed (panels a-e, k) or SEE-pulsed (panels f–j, l) Raji cells in the absence of Fc^#^ proteins and/or pre-treated with the indicated Fc^#^ protein (panels m-o) are shown; scale bar, 5 μm (panels a–j) and 10 μm (panels k–o). The percentage of Jurkat cells showing CD3 relocalization at the immunological synapse under both conditions (graph in the middle row), and the percentage of inhibition of CD3 relocalization in SEE-treated samples caused by each Fc^#^ protein relative to the samples without Fc^#^ protein (graph in the bottom row) are depicted. Data show the mean ± SD of six individual fields from two independent samples of a representative experiment out of two. At least 60 conjugates were analyzed per field. **(B)** Jurkat cells, pre-treated with 5 μg/mL of purified C5-Fc^#^, CD48-Fc^#^, CD58-Fc^#^, Ctl-Fc^#^, or left untreated, and co-cultured with unpulsed or SEE-pulsed Raji cells at a 1:1 ratio, were assessed after 16 h for IL-2 secretion by ELISA. IL-2 levels in cultures with unpulsed or SEE-pulsed Raji cells in the absence of Fc^#^ proteins are depicted (left panel). The percentage of IL-2 inhibition caused by the Fc^#^ proteins in SEE-treated co-cultures relative to the sample without any Fc^#^ protein is shown (right panel). Data represent the mean ± SD of three independent samples of a representative experiment out of three. Asterisks indicate statistically significance between SEE- and SEE+ samples or relative to Ctl-Fc^#^ or CD48-Fc^#^. **p <0.01, ***p<0.001, ****p<0.0001.

Given the established importance of CD58:CD2 interactions in T-cell synapse assembly, we examined whether C5-Fc^#^ disrupts antigen-dependent synapse formation. Conjugates were formed between Jurkat Vβ8^+^ T cells and Staphylococcus enterotoxin E (SEE)-pulsed Raji APCs and analyzed by immunofluorescence microscopy using anti-CD3 and anti-CD45 antibodies. In the absence of superantigen, conjugates were rare, and CD3 remained even and diffusely distributed across the Jurkat cell surface (**Figure 7A**, panels a-e, k, and graph in the middle row). SEE stimulation strongly increased the frequency of conjugates exhibiting well defined immunological synapses, characterized by CD3 accumulation at the T-B interface, whereas CD45 maintained a homogeneous cell surface distribution (**Figure 7A**, panels f-j, l, and graph in the middle row). Pre-exposure to C5-Fc^#^ substantially reduced the proportion of conjugates displaying CD3 polarization, with inhibition exceeding that of CD58-Fc^#^ and absent in Ctl-Fc^#^ (**Figure 7A**, panels m-o, and graph in the bottom row). To determine functional consequences, we quantified IL-2 production by Jurkat cells stimulated with SEE-loaded Raji cells. SEE induced robust IL-2 secretion (**Figure 7B**, left graph), whereas C5-Fc^#^ suppressed IL-2 release in a dose-dependent manner, similar to CD58-Fc^#^. Ctl-Fc^#^ and CD48-Fc^#^ proteins had no detectable effect (**Figure 7B**, right graph). These findings indicate that C5-Fc^#^ interrupts both structural assembly and functional output of antigen-dependent immunological synapse, providing a mechanistic basis for its inhibitory effects on T-cell activation.

### Identification of potential immunogenic hot spots in C5-Fc^#^

**Figure 8:**
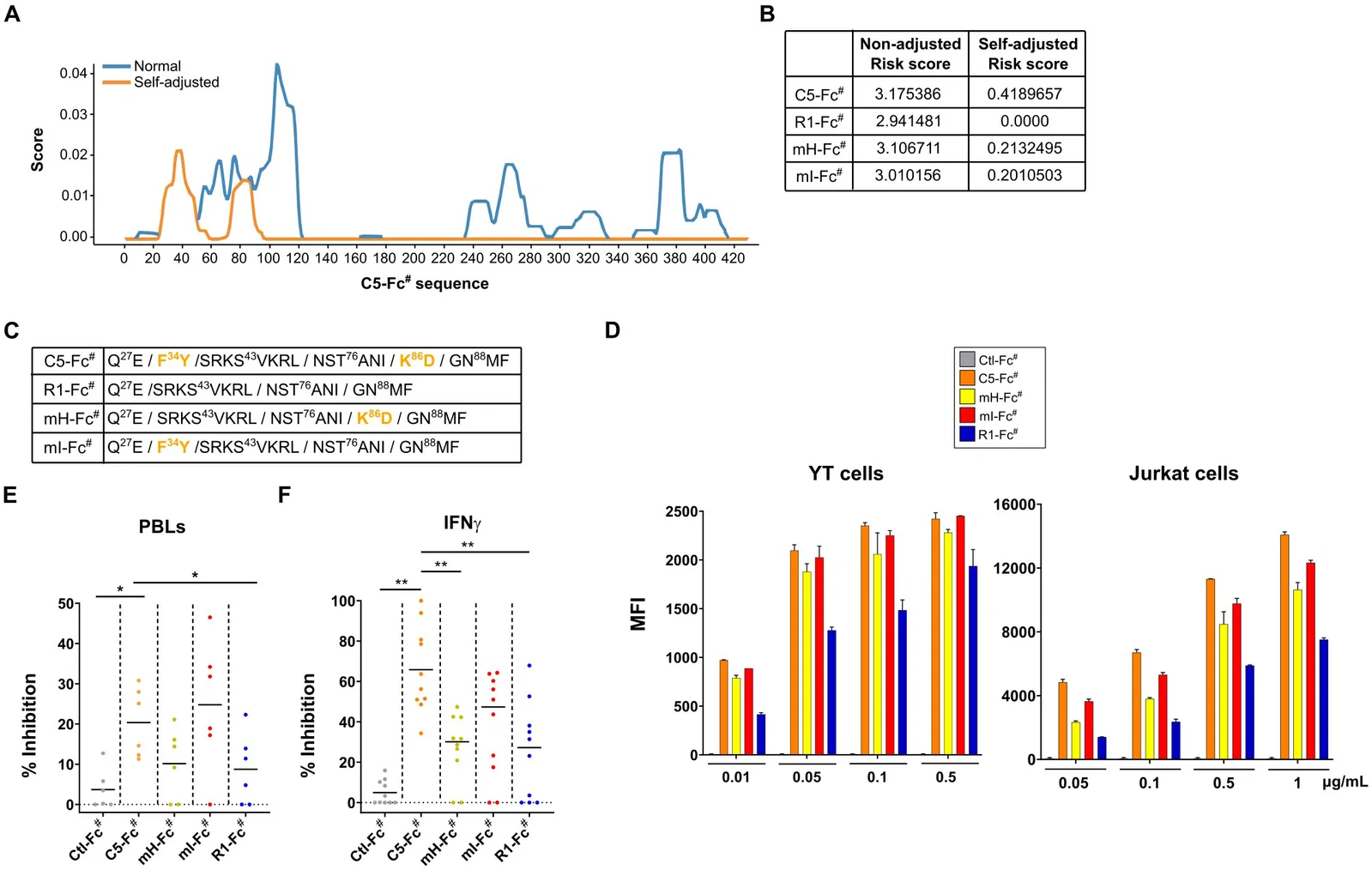
Generation and impact of CD48-Fc^#^ mutants devoid of predicted immunogenic hot spots on T-cell proliferation and IFNγ production in allogeneic MLRs. **(A)** Plot showing non-adjusted (blue) and self-adjusted (orange) immunogenicity risk scores along the C5-Fc^#^ sequence. **(B)** Table summarizing non-adjusted and self-adjusted risk scores for each mutant, calculated using the NetMCHIIpan prediction method. **(C)** Table showing mutations included in C5-Fc^#^, mH-Fc^#^, mI-Fc^#^, and R1-Fc^#^. Mutations F^34^Y and K^86^D are marked in orange. **(D)** YT and Jurkat cells were incubated with the indicated concentrations of purified C5-Fc^#^, mH-Fc^#^, mI-Fc^#^, R1-Fc^#^, or Ctl-Fc^#^, followed by anti-human IgG Fc-PE, and flow cytometry analysis. Shown are MFI values for the different interactions. Data are presented as the mean ± SD of duplicate samples from one representative experiment out of two. **(E)** CFSE-labeled responder PBLs were stimulated with irradiated allogeneic PBLs at a 1:1 ratio for 6 days in the absence or presence of 5 μg/mL of purified C5-Fc^#^, mH-Fc^#^, mI-Fc^#^, R1-Fc^#^ or Ctl-Fc^#^. CFSE-labeled responder PBLs stimulated with irradiated autologous PBLs in the absence of Fc^#^ proteins served as controls. Proliferation of total PBLs was quantified in six donor pairs by CFSE dilution via flow cytometry. Proliferation measured in the autologous co-culture controls was subtracted. The percentage of inhibition reflects reduced proliferation relative to these controls. Scatter dot plots show the mean across responder-stimulator pairs. **(F)** Co-cultures from 10 donor pairs (as in E) were analyzed on day 6 for IFNγ production by ELISA. Values were normalized to co-cultures of the same donor pair without Fc^#^ proteins, and percentage of inhibition calculated as the reduction of the IFNγ levels relative to these controls. Each symbol represents a donor pair and horizontal bars indicate the mean values. Asterisks denote statistically significant differences relative to C5-Fc^#^ exposed samples. *p<0.05, **p<0.01.

Although C5-Fc^#^ differs from human CD48 at only 12 positions, these substitutions could influence immunogenicity. We therefore assessed the immunogenic profile of C5-Fc^#^ using *in silico* prediction tools. Two potential immunogenic hot spots were identified when applying self-adjusted scoring, which incorporates tolerance to peptides present in the human proteome (**Figure 8A**). These regions mapped to residues 27–41 and 78–89 and contained segments YTYDQKIVE and IYIMRVLKD, encompassing replacements F^34^Y and K^86^D, respectively. Additional peaks were found between residues 100-120, and further in the sequence around 240–280 and 370-410, located in the Fc^#^ portion; however, the score risk for all three regions was eliminated upon self-adjustment. Consistently, the overall non-adjusted, and self-adjusted immunogenicity scores of C5-Fc^#^ were 3.175386 and 0.4189657, respectively (**Figure 8B**).

Taking into consideration the two potential immunogenic spots of C5-Fc^#^, we generated three new CD48-Fc^#^ variants in which residues Y^34^, D^86^ or both were reverted to the native CD48 sequence (**Figure 8C**). The resulting mutants, mH-Fc^#^ (Q^27^E/SRKS^43^VKRL/NST^76^ANI/K^86^D/GN^88^MF), mI-Fc^#^ (Q^27^E/F^34^Y/SRKS^43^VKRL/NST^76^ANI/GN^88^MF), and R1-Fc^#^ (Q^27^E/SRKS^43^VKRL/NST^76^ANI/GN^88^MF), showed reduced predicted immunogenicity. In particular, R1-Fc^#^ exhibited a self-adjusted risk score of 0, reflecting complete removal of the two potential hots pots regions (**Figure 8B**).

### Binding and functional properties of C5-Fc^#^ mutants lacking potential immunogenic hot spots

We examined the binding capacities of these three newly generated CD48-Fc^#^ mutants by measuring their interaction with YT and Jurkat cells by flow cytometry. As illustrated in **Figure 8D** (left panel), mH-Fc^#^ and mI-Fc^#^, which incorporated “back mutations” in only one of the two potential hot spots retained binding levels to YT cells comparable to C5-Fc^#^, particularly mI-Fc^#^, whereas R1-Fc^#^, in which both potential hot spots were reverted, displayed a decreased interaction across all tested concentrations. In assays performed in Jurkat cells (**Figure 8D**, right panel), R1-Fc^#^ also exhibited a substantial loss in interaction relative to C5-Fc^#^, mH-Fc^#^ showed a less pronounced decrease in binding, whereas mI-Fc^#^ maintained binding levels approaching those of C5-Fc^#^. As expected, Ctl-Fc^#^ showed negligible binding to CD244- or CD2-expressing cells, confirming specificity. Therefore, while reverting the aspartic acid at position 86 to the native lysine preserved C5-Fc^#^ binding to CD244 and CD2, substituting the tyrosine at position 34 with phenylalanine, and especially restoring both potential hot spots residues, led to a partial loss of binding to both receptors.

We next evaluated the functional competence of the three mutants in MLR assays. Consistent with the binding data, mI-Fc^#^ significantly reduced T-cell proliferation to an extent comparable to C5-Fc^#^, while mH-Fc^#^ and R1-Fc^#^ had weaker effects (**Figure 8E**). Analysis of IFNγ secretion across an expanded set of donor pairs yielded parallel results. mI-Fc^#^ markedly decreased cytokine production, mH-Fc^#^ and R1-Fc^#^ led to more modest effects, while the unrelated Ctl-Fc^#^ protein did not appreciably altered INFγ levels (**Figure 8F**). Thus, among the engineered mutants, ml-Fc^#^ mostly maintains both receptor engagement and immunomodulatory activity, positioning it as an improved, less immunogenic derivative of C5-Fc^#^.

## Discussion

Large DNA viruses such as herpesviruses and poxviruses have developed elaborated immune evasion strategies, including the expression of host-mimicking molecules that modulate immune responses to promote viral replication and persistence. A43, a herpesvirus-encoded CD48 homolog, exemplifies this strategy by functioning as a soluble decoy receptor that binds CD244 and, as shown here, can also interact with CD2 (23). Motivated by this viral mechanism, we engineered soluble human CD48 mutants capable of modulating these two co-signaling pathways in NK and T cells. This work highlights how viral immune-evasion strategies can serve as conceptual templates for developing next-generation immunomodulatory therapeutics intended to restore immune balance in inflammatory and autoimmune diseases (30).

To achieve this goal, CD48 mutants were generated as Fc-fusion proteins containing the two extracellular domains of human CD48 linked to a human IgG1 Fc region. This format offers important advantages, including enhanced solubility, structural stability, and extended serum half-life, thereby facilitating both mechanistic studies and potential therapeutic use. Equally important, the bivalent architecture of Fc-fusion proteins increases functional avidity for target ligands, enabling more effective blockade of receptor-ligand interactions (31). Such constructs can be strategically designed to interfere with co-stimulatory pathways that regulate leukocyte activation, and thereby attenuate pathogenic immune signaling. This principle has been validated clinically; for instance, abatacept, a CTLA-4-IgG1 fusion protein with a silenced-Fc domain, suppresses T-cell co-stimulation by outcompeting CD28 for CD80/CD86 binding, and is approved for multiple autoimmune conditions including rheumatoid arthritis, psoriatic arthritis, and juvenile idiopathic arthritis, as well as prevention of acute graft-versus-host disease (32–36). Similarly, alefacept, a CD58-IgG1 fusion protein that was used for psoriasis, modulates the CD2 axis by simultaneously blocking CD2 and inducing FcγR-mediated depletion of CD2-high effector memory T cells (37, 38). Unlike alefacept, our CD48-based human IgG1 proteins contain a silenced Fc domain (Fc^#^) to avoid lymphocyte depletion and are designed to provide a dual blockade of CD2 and CD244, thereby enhancing immunomodulatory potency.

Among the engineered variants, C5-Fc, the variant incorporating 12 amino acid substitutions, displayed the strongest enhancement in binding to both CD244 and CD2. This increased binding was observed using COS-7 cells ectopically expressing each receptor, as well as in NK (YT) and T (Jurkat) cell lines endogenously expressing CD244 or CD2. Binding was strictly specific, as no interaction occurred with other SLAM family members. Additionally, C5-Fc bound soluble CD244 and CD2 in a dose-dependent manner. SPR analyses further demonstrated that C5-Fc possesses markedly increased avidity for CD244 (KD 0.2 nM), primarily due to a dissociation rate 16.5-fold slower than that of wild-type CD48-Fc. C5-Fc also bound CD2 with a KD of 62 nM, close to the affinity of CD58-Fc (23 nM). These KD values reflect avidity effects arising from Fc-mediated bivalency and thus differ from published monomeric affinity data (8, 29). The congruence between cell-based binding, ELISA, SPR, and structural modelling strongly supports the conclusion that C5-Fc displays substantially improved binding kinetics and functional avidity.

Structural modeling clarifies how specific mutations contribute to reinforce interactions without causing major structural perturbations. The Q^27^E substitution is positioned centrally within the binding interface and proved critical for improved binding to both CD244 and CD2. This glutamine-to-glutamate change introduces new electrostatic interactions with K^47^ in CD244 and K^34^/K^43^ in CD2. Consistent with this model, the Q^27^A mutant, which lacks the capacity to engage in such charge-based interactions, did not enhance binding. Another mutation with significant cooperative effects is F^34^Y. Although the single F^34^Y substitution did not improve binding (mutant S2-Fc), reverting this mutation within the C5-Fc^#^ background (mH-Fc^#^) diminished binding, especially to CD2. In the structural context of C5-Fc, the introduced tyrosine at position 34 not only preserves the aromatic stacking profile of phenylalanine but also adds a hydroxyl group that enables new hydrogen-bonding contacts. This additional polarity allows Y^34^ to engage V^95^ and T^97^ in CD244 and to form stabilizing interactions with Y^86^, N^92^, and E^95^ in CD2, thereby augmenting affinity when local geometry is available. The GN^88^MF substitution also contributes substantially, as these residues form extensive non-bonding interactions with both CD244 and CD2 and, when introduced alone (mutant A3-Fc), significantly enhance binding, particularly to CD244.

Other substitutions in C5-Fc likely act by indirectly enhancing binding via local structural effects. For example, the SRKS^43^VKLR replacement occurs within the C’C’’ loop and interacts weakly with CD2 but not CD244. Nonetheless, the marked difference between C4-Fc and C5-Fc, which differ only at this site, indicates a cooperative contribution when combined with other mutations. The K^86^D substitution lies outside the direct binding interface and does not alter binding when introduced alone (S3-Fc), yet its reversion in C5-Fc^#^ slightly reduced CD2 affinity (mI-Fc^#^), suggesting a modest indirect role. Finally, the NST^76^ANI mutation removes a glycosylation site preceding β-strand F and appears to enhance CD2 binding, as supported by the A2-Fc mutant. Collectively, these observations indicate that C5-Fc benefits from a combination of direct enhancements in non-bonding contacts and indirect improvements arising from conformational stabilization and optimized packing at the binding interface.

The structural modeling also predicted that the CD244- and CD2-binding interfaces on C5 substantially overlap, suggesting that engagement of these receptors is likely mutually exclusive. Given that C5-Fc binds CD244 with higher affinity than CD2, receptor occupancy is expected to be determined by both receptor expression levels and binding affinity. Consequently, C5-Fc^#^ is predicted to preferentially bind CD244 on NK cells, whereas CD2 binding is likely favored in T-cell populations that express low levels of CD244 or lack CD244 expression altogether.

Functionally, C5-Fc effectively outcompeted native CD48 and CD58 interactions with CD244 and CD2 in both cellular- and solution-based assays. Notably, C5-Fc blocked the interaction of CD58 with CD2 expressed on the cell surface more potently than CD58-Fc, despite CD58-Fc showing higher binding to CD2. C5-Fc^#^ also efficiently disrupted NK-cell conjugate formation with CD48-expressing targets and T-cell conjugates with CD58-expressing cells, confirming its robust capacity to interfere with CD244- and CD2-dependent cell-cell interactions.

Given the central roles of CD48:CD244 and CD58:CD2 interactions in NK-cell activation and effector responses (16, 39), we assessed the effect of C5-Fc^#^ in NK-cell cytotoxicity assays using models selectively dependent on either CD244 or CD2. C5-Fc^#^ significantly reduced NK cell-mediated killing in both systems, outperforming CD48-Fc^#^ and CD58-Fc^#^. The inhibitory effect observed in the CD2-dependent assay was more modest, likely reflecting the low level of CD2 expression on the NK-92 effector cells used. This is particularly relevant because no approved immunotherapies specifically target NK-cell pathways in autoimmunity despite substantial evidence implicating NK cells in disease pathogenesis. NK cells infiltrate the pancreas in type 1 diabetes and contribute to β-cell destruction (40), promote keratinocyte damage and amplify inflammation in psoriasis (41), and participate in epithelial injury in Sjögren’s syndrome (42, 43). These pathogenic activities involve both cytotoxic mechanisms and the production of inflammatory cytokines such as IFNγ (44). Thus, the ability of C5-Fc^#^ to simultaneously restrain CD244- and CD2-dependent activation presents a unique opportunity to modulate NK-cell-driven pathology.

Consistent with the role of CD244 and CD2 as co-receptors, C5-Fc^#^ had only a limited effect on NK cell-mediated ADCC, with no significant inhibition observed for most target cell populations. These findings suggest that blockade of the CD244:CD48 and CD2:CD58 pathways is insufficient to overcome the strong activating signals driven by CD16 engagement and indicate that C5-Fc^#^ largely preserves Fc receptor-dependent cytotoxic responses.

Because CD2 is expressed broadly on T cells and CD244 is upregulated on effector cytotoxic T cells, we also analyzed the effect of C5-Fc^#^ on T-cell activation using an MLR assay. C5-Fc^#^ inhibited both CD4^+^ and CD8^+^ T-cell proliferation and significantly reduced IFNγ and TNFα secretion. Mechanistically, C5-Fc^#^ impaired the maturation of the immunological synapse, evidenced by diminished stable T-APC conjugates with CD3 polarization. These defects likely reflect interference with early co-stimulatory signals required for full T-cell activation, and they explain the broad inhibitory effects observed in proliferation and cytokine-production assays. By targeting two major co-signaling pathways simultaneously, C5-Fc^#^ acts as an early blocker of lymphocyte activation, underscoring its potential as a therapeutic agent capable of dampening hyperactive or autoreactive immune responses.

Although SAP deficiency can profoundly alter CD244 signaling by converting it from an activating to an inhibitory receptor, there is little evidence that SAP-deficient lymphocytes are a prominent feature of autoimmune diseases. In contrast, SAP-expressing pathogenic T-cell subsets, including peripheral helper T cells, have been reported in patients with lupus nephritis (45).

Notably, C5-Fc^#^ significantly inhibited T cell-mediated cytotoxicity in a redirected killing assay. Together with its effects on lymphocyte activation, these findings indicate that C5-Fc^#^ can modulate cytotoxic immune responses and may regulate pathogenic T-cell activity in immune-mediated diseases. Further studies are needed to define the mechanisms involved.

An important challenge in translating viral homologs into therapeutic proteins is minimizing immunogenicity. Viral proteins are recognized as foreign antigens and may elicit neutralizing antibodies or inflammatory reactions. The substantial sequence divergence between A43 and human CD48 (66% ectodomain identity) raised concerns about immunogenicity. This motivated our strategy of engineering CD48-based variants with minimal A43-guided substitutions, thereby improving functional properties while maintaining high sequence identity to human CD48. C5-Fc^#^ incorporates only 12 substitutions, less than 3% of the protein sequence, far fewer than the modifications typically involved in humanized monoclonal antibodies.

An *in silico* immunogenicity assessment centered on MHC class II-restricted epitopes, which represent the primary focus of immunogenicity risk for therapeutic proteins, identified two potential hotspots in C5-Fc^#^: F^34^Y and K^86^D. Although evaluation of MHC class I-restricted epitopes could provide complementary information, it was beyond the scope of the present study and is unlikely to affect the overall interpretation of the immunogenicity risk of C5-Fc^#^. We therefore generated three additional C5-Fc^#^ variants in which F^34^ (mH-Fc^#^), K^86^ (mI-Fc^#^) or both residues (R1-Fc^#^) were reverted, and evaluated their binding and functional properties. These reversions partially reduced CD2 binding to distinct extents while largely preserving CD244 affinity. Consistently, MLR assays showed graded decreases in immunomodulatory potency, with mI-Fc^#^ retaining the highest activity, followed by mH-Fc^#^ and finally the double revertant R1-Fc^#^. Thus, while reversion of selected residues affected binding to varying degrees, mI-Fc^#^ maintained both receptor binding and functional activity comparable to C5-Fc^#^ across the concentration range relevant for biological activity, indicating that reduced predicted immunogenicity can be achieved without compromising efficacy.

Although *in silico* predictions cannot fully capture the complexity of human immune responses, the lower predicted scores combined with preserved functional capacity support the feasibility of minimal engineering to balance therapeutic efficacy and immunological safety.

A major limitation of this study is that all findings were generated *in vitro*. Consequently, the efficacy, pharmacokinetics, tissue distribution, and safety of C5-Fc^#^ remain to be established *in vivo*, and its translational potential should therefore be interpreted with caution. Future studies in relevant preclinical models, including humanized mice, will be needed to evaluate therapeutic efficacy, effects on disease-associated immune responses, and safety, as well as to define the pharmacokinetic and pharmacodynamic properties required for clinical development.

In conclusion, this work demonstrates how insights from viral immune evasion can guide rational engineering of host molecules to create effective and selective immunomodulators. The engineered CD48 variants C5-Fc^#^ and mI-Fc^#^ combine enhanced dual targeting of CD244 and CD2 with minor sequence modification, potent inhibition of NK- and T-cell responses, and a favorable predicted immunogenicity profile. These features highlight their promise as novel therapeutic candidates for modulating pathological immune activation in autoimmune and inflammatory diseases.

## Materials and methods

### Cell culture

COS-7, HEK-293T, Jurkat, YT, K562, and Raji cell lines were obtained from the American Type Culture Collection (Manassas, VA, USA). The NK-92 line was provided by T. Bellón (La Paz University Hospital Health Research Institute, Madrid, Spain). The Jurkat-derived human cell line Vβ8^+^ J77 cl20 was provided by A. Alcover (46). COS-7 and HEK-293T cells were cultured in Dulbecco’s modified Eagle’s medium (DMEM; Cytiva, Malborough, MA, USA), and Jurkat, K562, Raji, and NK-92 cells in RPMI-1640 medium (Corning, NY, USA). All media were supplemented with 2 mM glutamine (Gibco, NY, USA), 1 mM sodium pyruvate (Gibco), 50 U/mL of penicillin, 50 μg/mL of streptomycin (Corning), and 10% fetal bovine serum (FBS; Pan Biotech, Aidenbach, Germany). NK-92 cultures additionally contained 200 U/mL rhIL-2 (ImmunoTools, Friesoythe, Germany) and 5% human AB serum (Sigma-Aldrich, Burlington, MA, USA). K562 cells stably expressing CD48 were maintained in 1.2 mg/mL of G418 (Gibco). Human peripheral blood mononuclear cells (PBMCs) were isolated from buffy coats using Histopaque (Sigma-Aldrich) and maintained in complete RPMI-1640.

### Antibodies

Mouse anti-human CD244 (clone 305), anti-human CD319 (clone 3.14A), and anti-human IgG (clone 29.5.5) antibodies were produced in-house (42, 47). Goat anti-human IgG-PE (Fc-specific) and anti-mouse IgG-PE (Fc-specific) antibodies for flow cytometry were from Jackson ImmunoResearch (Philadelphia, PE, USA). Mouse anti-human CD2-PE (clone TS1/8), anti-human CD84-PE (clone CD84.1.21) and anti-human CD3-PE (clone UCHT1) were from EXBIO (Vestec, Czech Republic). Anti-human IgG-POD (Fc-specific), anti-mouse IgG (Fc-specific), and anti-mouse IgG γ-chain specific-POD antibodies for ELISA were from Sigma (Burlington, MA, USA). Mouse anti-human CD48-PE (clone TÜ145), anti-human CD58-PE (clone L306.4), anti-human CD8-Alexa Fluor 647 (clone RPA-T8), anti-human CD4-PE (clone RPA-T4), anti-human CD3-BV421 (clone SK7), anti-human CD4-FITC (clone L200), and anti-human CD8-PE-Cy5 (clone RPA-T8) antibodies were from BD Biosciences (San Jose, CA, USA). Antibody anti-human CD52 alemtuzumab (clone Campath-1H) was from Leinco Technologies (St Louis, MO, USA). The Blinatumomab Biosimilar antibody (anti-human CD3 x anti-human CD19 bispecific antibody) was from Proteogenix (Schiltigheim, France). Mouse anti-human CD229-PE (clone HLy-9.1.25), anti-human CD352-PE (clone NT-7) and anti-human CD45-Alexa Fluor 488 (clone HI30) were from BioLegend (San Diego, CA, USA). Mouse anti-human CD150-PE antibody (clone SLAM.4) was from Abcam (Cambridge, United Kingdom/UK).

### Plasmid constructions

Plasmids pCI-neo CD48, pCI-neo CD2, pCI-neo CD244, pCI-neo CD84, pCI-neo CD150, pCI-neo CD229, pCI-neo CD352, and pCI-neo CD319, encoding the corresponding full-length proteins in pCI-neo (Promega, Madison, WI, USA) were previously described (48). Plasmids pCI-neo A43-Fc, pCI-neo CD48-Fc and pCI-neo CD244-Fc, encoding the two Ig domains of the corresponding molecules fused to the Fc-region of human IgG1, were also reported (23, 47). Mutant CD48-Fc plasmids (pCI-neo CD48 S1-Fc, pCI-neo CD48 S2-Fc, pCI-neo CD48 S3-Fc, pCI-neo CD48 S4-Fc, pCI-neo CD48 A1-Fc, pCI-neo CD48 A2-Fc, pCI-neo CD48 A3-Fc, pCI-neo CD48 C4-Fc, and pCI-neo CD48 C5-Fc), were generated by Splicing by Overlap Extension (SOE)-PCRs using pCI-neo CD48-Fc as template, except for pCI-neo CD48 C4-Fc which was produced through sequential SOE-PCR rounds with pCI-neo CD48-Fc templates in which mutations were introduced sequentially, and pCI-neo CD48 C5-Fc where pCI-neo CD48 C4-Fc was the template employed. In these SOE-PCRs, the same external primers hCD48For and hCD48Rev with BamHI restriction sites were used, along with specific internal primers carrying the required mutations. The resulting PCR products were cloned into pGEM-T (Promega), excised with BamHI, and ligated in-frame into BamHI-digested pCI-neo Fc plasmid (23). Plasmids pCI-neo CD58-Fc and pCI-neo CD2-Fc, encoding the two Ig domains of human CD58 and CD2, respectively, fused to the Fc region of human IgG1, were obtained by PCR from pCI-neo CD58 or pCI-neo CD2 using primers with BamHI restriction sites, cloned into gem pGEM-T, and transferred to BamHI-digested pCI-neo Fc. Plasmid pFUSE CD48-Fc, expressing the two Ig domains of human CD48 fused to the Fc region of murine IgG1, was generated by PCR from pCI-neo CD48 employing specific primers containing EcoRI and BglII restriction sites, inserted into pGEM-T and cloned into the EcoRI/BglII-digested pFUSE-mIGg1-Fc vector (InvivoGen, San Diego, CA, USA). Plasmid pFUSE CD58-Fc, encoding the two Ig domains of human CD58 fused to the Fc region of murine IgG1, was constructed as indicated for pFUSE CD48-Fc, except that a SOE-PCR was performed to eliminate the EcoRI site in pCI-neo CD58-Fc before amplification of the CD33 signal peptide and Ig domains. Oligonucleotides used for cloning are listed in **Supplementary Table 1**. All recombinant plasmids were verified by DNA sequencing and, when necessary, by restriction enzyme digestion. PCR conditions were: 1 cycle at 94 °C for 5 min; 30 cycles of 1 min at 94 °C, 1 min at 51 °C, and 1 min at 72 °C; and 1 cycle at 72 °C for 10 min. Conditions for annealing reactions were: 6 cycles of 5 min at 94 °C; 1 cycle at 51 °C for 1 min; 1 cycle at 72 °C for 1 min; and 1 cycle of 10 min at 72 °C.

### Transfections and generation of Ig fusion proteins

COS-7 cells were transiently transfected employing the Cell Line Nucleofector Kit R solution and the Amaxa Nucleofector I/II/2b device (Lonza, Basel, Switzerland). Human IgG1 (-Fc) and mouse IgG 1 (-mFc) fusion proteins were produced by transiently transfecting HEK-293T cells with polyethylenimine in Opti-MEM medium (Gibco), as previously described (23). Supernatants were collected 5–7 days later, centrifuged at 1500 x g for 10 min, and concentrated using Amicon Ultra Centrifugal filters (Millipore, MA, USA). Fc-fusion proteins were quantified by ELISA. Purified Fc- and Fc^#^-fusion proteins, containing the two Ig domains of the indicated molecules fused to the Fc region or a silent version of the Fc region of human IgG1 designated here as Fc^#^, were obtained from GenScript (New Jersey, USA). The Fc^#^ variant includes mutations L^234^A and L^235^A to reduce antibody-dependent cell-mediated cytotoxicity and complement-dependent cytotoxicity, together with mutations M^252^Y, S^254^T, and T^256^E to extend their half-life, of human IgG1 (49, 50). These purified Fc- and Fc^#^-fusion proteins were produced using Turbo-CHO platform, and purity confirmed by SDS-PAGE and size-exclusion chromatography high performance liquid chromatography (SEC-HPLC) (>95%; GenScript). SEC-HPLC profiles of the purified Fc-fusion proteins are shown in **Supplementary Figure 4**.

### Flow cytometry assay

Flow cytometry was performed following standard procedures (51), using PBS containing 20% rabbit serum (Linus, Surrey, Canada) and 1% of FBS. Cells were incubated with the appropriate antibody and, when needed, detected using an anti-human IgG-PE (Fc-specific). Staining with Fc- or Fc^#-^ fusion proteins was followed by anti-human IgG-PE (Fc-specific) detection, and using an irrelevant Fc- or Fc^#^-fusion protein (Ctl-Fc) as a negative control. Non-specific binding was assessed using anti-human IgG-PE only. For blocking assays, YT cells or Jurkat cells were incubated with purified Fc-fusion proteins, and subsequently incubated with CD48-mFc or CD58-mFc, respectively, followed by anti-mouse IgG PE (Fc-specific). Cells were acquired on FACSCanto II, LSRFortessa 4L, or LSRFortessa 5L (BD Biosciences) and analyzed using FlowJo Xv10.0.7 software (Tree Star, Inc, BD Biosciences).

### Enzyme-linked immunosorbent assay

Ninety-six-well plates were coated with 2-3 μg/mL per well of protein and blocked with 1% BSA (Capricorn Scientific, Ebsdorfergrund, Germany) in PBS. Fc-fusion proteins from supernatants of HEK-293T transfected cells were quantified by sandwich ELISA employing immobilized anti-human IgG Fc and anti-human IgG-POD (detection) antibodies, with purified human IgG1 as a standard. A similar ELISA with immobilized His-tagged CD244 or CD2 proteins (Sino Biological, Beijing, China) was used to assess binding of purified Fc-fusion proteins. For competition ELISAs, immobilized purified CD244-Fc or CD2-Fc proteins were incubated with varying concentrations of purified Fc protein, followed by CD48-mFc or CD58-mFc, respectively, and detection with anti-mouse IgG-POD antibody. Human IL-2 DuoSet, IFN-gamma DuoSet and TNF-alpha DuoSet ELISA kits (Bio-Techne R&D Systems, Minneapolis, MN, USA) were employed according to the manufacturer’s instructions. Reactions were developed with TMB substrate (BD Biosciences), stopped with 0.5 M H_2_SO_4_, and absorbance measured at 450/570 nm using a Multiskan FC microplate reader (Thermo Scientific, Waltham, MA, USA).

### SPR analyses

SPR experiments were conducted at 25 °C on a Biacore 8K instrument (Cytiva). Approximately 20 RU of human His-tagged CD244 and CD2 proteins (ACROBiosystems, Beijing, China) were captured on a Series S CM5 sensor chip (Cytiva) via anti-histidine antibodies. Serial twofold dilutions of purified Fc proteins (seven to nine concentrations ranging from 0.390625 nM to 100 nM, depending on the interaction) were injected at 30 μL/min for 90 s, followed by a 120 s dissociation phase in HBS-EP at the same flow rate. Sensor chip surfaces were regenerated with 10 mM glycine-HCl (pH 1.5) at 30 μL/min for 60 s. Kinetic parameters were determined using Biacore Insight Evaluation Software (v5.0.18.22102), fitting sensorgrams to 1:1 binding model.

### Conjugate formation assay

Effector cells (YT or Jurkat) and target cells (K562 or K562 CD48) were stained with CellTracker Blue CMAC (10 μM, Invitrogen, Carlsbad, CA, USA) and CellTrace CFSE (1.25 μM, Invitrogen), respectively, for 20 min at 37 °C in serum free media. Labeled cells were washed, rested for 5 min at 37°C and resuspended in complete medium. A total of 1x10^5^ effector cells were co-incubated with target cells at a 1:1 effector-to-target cell (E:T) ratio in the absence or presence of 5 μg/mL of the indicated purified Fc^#^-fusion protein at 37°C for 0 or 20 min, and subsequently placed on ice. Cells were analyzed by flow cytometry, with double-positive events scored as conjugates.

### Immunological synapse formation assay

Raji cells were CMAC-labeled as indicated above and pulsed with SEE (2 μg/mL, Toxin Technology, Saratosa, FL, USA) for 20 minutes at 37°C. Vβ8^+^ Jurkat cells, pre-treated with 5 μg/mL of Fc^#^-fusion proteins for 30 min at 37°C, were mixed with labeled Raji cells at an E:T ratio of 1:1 for 20 min. Cell mixtures were plated on poly-L-lysine-coated (0,01%, Sigma) μ-slide 8 well high chambers (IBIDI, Gräfelfing, Germany), fixed in 2% paraformaldehyde for 10 min, blocked with 6% FBS in PBS, and stained with anti-CD3-PE and anti-CD45-Alexa Fluor 488. Images were acquired using a Leica Thunder microscope (Leica Microsystems) with a Leica PL APO 63x numerical aperture 1.4 oil immersion. Image acquisition was performed using the software LasX Navigator. Automated image analysis was performed using custom-made ImageJ macros implemented in Fiji/ImageJ (NIH) to quantify immune cell interactions and polarization in multi-channel microscopy images. The workflow included supervised machine-learning–based segmentation of brightfield images using the Trainable Weka Segmentation plugin, classification of effector and target cells, and identification of cell–cell contact regions via morphological operations. Fluorescence intensities were quantified at both whole-cell and contact-interface levels and per-cell and summary metrics were automatically exported.

### NK cell cytotoxicity assay

YT and NK-92 cell cytotoxicity was assessed by the calcein-AM release assay (52). Target cells (K562 or K562-CD48) were labeled with 2 μg/mL calcein-AM (Invitrogen) for 30 min at 37°C in serum-free medium. After washing, 5x10^3^ labeled target cells per well were incubated with effector cells for 4 h at 37 °C at various E:T cell ratios in V-bottom 96-well plates. When indicated, effector cells were pre-incubated with 5 μg/mL of Fc^#^-fusion proteins, for 30 min at 37 °C. Maximum and spontaneous release controls were obtained by treating target cells for 4 h with 1% Triton X-100 or plain medium, respectively. Medium alone, without cells, served as a background control. Cell-free culture supernatants were transferred to 96-well flat-bottom plates and fluorescence measured on a Spark multimode microplate reader (Tecan, Männedorf, Germany). Lysis percentage was calculated as (experimental release–spontaneous release)/(maximum release–spontaneous release)x100. Background fluorescence was subtracted from all samples.

### ADCC assay induced by anti-CD52.

PBMCs were used as target cells, and NK cells, isolated from autologous PBMCs using the Human NK Cell Isolation Kit (Miltenyi Biotec, Bergisch Gladbach, Germany), were used as effector cells. Target and effector cells were co-cultured at an E:T ratio of 2:1 in the presence of the ADCC-inducing anti-human CD52 monoclonal antibody alemtuzumab at 1 µg/mL and 5 µg/mL of the indicated purified Fc^#^ fusion proteins at 37°C in round-bottom 96-well culture plates. After 4 h of incubation, lysis of target T-cell subsets was quantified by flow cytometry using Fixable Viability Dye eFluor 506 (Thermo Fisher Scientific) in combination with anti-human CD3-BV421, anti-human CD4-FITC, and anti-human CD8-Pe-Cy5 monoclonal antibodies.

### Mixed lymphocyte reaction (MLR) assay

PBMCs isolated from buffy coats were used in pairwise combinations. Irradiated PBMCs (30 Gy) from one donor served as stimulator cells and PBMCs from another donor, labeled with 2.5 μM CellTrace CFSE, served as responder cells. Stimulator and responder cells (1x10^5^ each per well) were co-cultured in round-bottom 96-well culture plates, in the absence or presence of 5 μg/mL of purified Fc^#^-fusion proteins for 6 days at 37°C. Cell-free culture supernatants were analyzed for IFNγ and TNFα by ELISA. Responder proliferation was determined based on CFSE signal dilution by flow cytometry. When indicated, CD4^+^ and CD8^+^ T-cell proliferation was assessed after staining with anti-CD4-PE and anti-CD8-Alexa Fluor 647.

### Redirected T cell cytotoxicity assay

1x10^4^ Raji target cells, labeled with 1 μM CellTrace Far Red (Thermo Fisher Scientific), were co-cultured with PBMC effector cells at an E:T ratio of 10:1. Cells were incubated in round-bottom 96-well culture plates in the presence of 0.1 ng/mL blinatumomab biosimilar (anti-CD3 × anti-CD19 bispecific antibody) and 5 μg/mL of the indicated purified Fc^#^ fusion proteins for 24 h at 37 °C. The bispecific antibody redirects endogenous T cells toward CD19-expressing Raji cells, thereby inducing T-cell-mediated cytotoxicity independently of native TCR specificity. After 24 h, the cells were harvested and Raji cell cytotoxicity was determined by the decrease in CellTrace positive cells by flow cytometry.

### Structural modeling and energetic evaluation of complexes

The structure of CD48:CD244, C5:CD244, CD48:CD2, and C5:CD2 complexes were obtained by comparative modeling using M4T (53) as follows. For CD48:CD244 and C5:CD244 complexes, the structures of human CD48 (PDB code: 2edo) and mouse CD48:CD244 complex (27) were used as templates. For CD48:CD2 and C5:CD2 complexes, the templates from human CD48 (PDB code: 2edo) and CD58:CD2 (54) (PDB code 1qa9) were combined. A total of 10 different models were generated for each complex. Binding energies of the protein complexes were computed using ZRANK (55). ZRANK scoring function is based on a linear combination of weighted physics-based metrics that include electrostatics, van der Waals and desolvation terms. A statistically significant association between ZRANK score distributions and binding affinities across interface residue mutants involved in protein-protein interactions was previously established (56).

### Immunogenicity prediction of CD48 mutants

The prediction of immunogenicity for the different sequences was performed using NetMCHIIpan v4.3 (57), a machine-learning prediction method based on Neural Network models trained using MHC-II immunopeptidomics. Each sequence variant was scanned against a set of 49 HLA-II (**Supplementary Table 2**) as overlapping 13 to 19-mer peptides to compute an immunogenicity score at a given residue position. The score of a given peptide was considered a potential epitope if the predicted rank value was less than or equal to 1% as previously reported (58, 59). Besides position specific individual scores, two global scores were computed for each sequence: a non-adjusted and a self-adjusted risk score. The non-adjusted risk score is computed as the sum of scores of identified potential epitopes weighted by the frequency of the given allele at the World population. The frequency of each allele considered was taken from the Allele Frequencies database (60). The self-adjusted risk scores are derived from non-adjusted scores by taking into consideration whether the potential epitope is present in the human proteome. Thus, self-adjusted scores account for self-tolerance adjusting the score to 0 if the predicted binding core of the epitope is present in the human proteome. The human proteome used for this analysis was obtained from the Uniprot database release 2025_02 (https://uniprot.org).

### Statistical analyses

Statistical analyses were performed in GraphPad Prism v10 (San Diego, CA, USA). Statistical significance between two groups was determined by a two-tailed unpaired t-test, except for the MLR assays, for which comparisons were conducted using the non-parametric Wilcoxon signed-rank. A p-value < 0.05 was considered statistically significant. Results are presented as mean ± standard deviation (SD).

## Data Availability

The original contributions presented in the study are included in the article/**Supplementary Material**. Further inquiries can be directed to the corresponding author.
