# Supplementary material for: Dual targeting of CD244 and CD2 by a viral-immunoevasin-guided engineered CD48-Fc mutant for T- and NK-cell modulation
Source: Front Immunol. 2026 Sep 1;17:1876221. doi: 10.3389/fimmu.2026.1876221 (PMC13575745; doi:10.3389/fimmu.2026.1876221)
Supplement: Supplementary file 1 [file DataSheet1.docx]

Supplementary Material

# 1 Supplementary Figures and Tables

## 1.1 Supplementary Figures

**Supplementary figure 1. Reactivity of A43-Fc and C5-Fc with members of the SLAM family.**

COS-7 cells non-transfected (left panels) or transfected with the indicated SLAM receptor (four right panels) were analyzed by flow cytometry to assess the expression of the corresponding receptors (two left panels), and their interaction with 5 μg/mL of A43-Fc, C5-Fc or Ctl-Fc (three right panels) using an anti-human IgG Fc-PE antibody. Shown are representative histograms and MFI values out of duplicate samples from two independent experiments. Dashed-line histograms refer to cells incubated with anti-human IgG Fc-PE antibody only.

**Supplementary figure 2. Structural comparison between CD48 and the C5 variant.**

CD48 is shown in blue color and cartoon representation while structural models of C5 variants from 10 independent simulations are shown in grey and ribbon traces. Dashed boxes indicate regions enlarged in the surrounding panels to highlight conformational changes between the two structures. The upper panels show representative differences in surface-exposed loops, whereas the lower panels focus on conformational rearrangements near the N-terminal (Nt) and C-terminal (Ct) regions. Residues changed in C5 variants are indicated and shown as sticks. Nt and Ct denote the N- and C-terminal regions, respectively.

**Supplementary figure 3. Impact of C5-Fc^#^ on NK cell-mediated ADCC.**

Autologous NK cells isolated from PBMCs were co-cultured with PBMC target cells at an E:T ratio of 2:1 in the presence of 1 μg/mL of alemtuzumab and 5 μg/mL of the indicated purified Fc^#^ fusion proteins. After 4 h, target cell lysis was assessed by flow cytometry using the Fixable Viability Dye eFLuor 506 and antibodies against CD3, CD4, and CD8 to quantify the susceptibility of T-cell subsets to NK cell-mediated ADCC. Data represents the mean ± SD of triplicate samples from one out of two independent experiments. Asterisks mark statistically significant differences relative to samples unexposed to purified Fc^#^-fusion proteins. *p<0.05, **<0.01.

**Supplementary figure 4. SEC-HPLC analysis of purified recombinant Fc-fusion proteins.**

SEC-HPLC analysis of the Fc-fusion proteins: CD48-Fc (A), CD58-Fc (B), C4-Fc (C), C5-Fc (D), CD48-Fc^#^ (E), CD58-Fc^#^ (F), C5-Fc^#^ (G), Ctl-Fc^#^ (H), mH-Fc^#^ (I), mI-Fc^#^ (J), and R1-Fc^#^ (K).

## 1.2 Supplementary tables

| **Plasmid name** | **Oligonucleotide name** | **Oligonucleotide sequence (5’**🡪**3’)** |
| --- | --- | --- |
| pCI-neo CD48 S1-  Fc | SOEQ27EhCD48Rev | AAAACCAGGTTAGTTCTTTGTAGTTCTCAGG |
|  | SOE Q27EhCD48For | CCTGAGAACTACAAAGAACTAACCTGGTTTT |
| pCI-neo CD48 S2-  Fc | SOEF34YhCD48Rev | ACAATCTTCTGGTCGTAAGTATAAAACCAGG |
|  | SOEF34YhCD48For | CCTGGTTTTATACTTACGACCAGAAGATTGT |
| pCI-neo CD48 S3-  Fc | SOEK86DhCD48Rev | TTGCTCATTCCCAGTGTCTTTCAACACCCTCAT |
|  | SOEK86DhCD48For | ATGAGGGTGTTGAAAGACACTGGGAATGAGCAA |
| pCI-neo CD48 S4-  Fc | SOEQ27AhCD48Rev | TAAAACCAGGTTAGTGCTTTGTAGTTCTCAGG |
|  | SOEQ27AhCD48For | CCTGAGAACTACAAAGCACTAACCTGGTTTTA |
| pCI-neo CD48 A1-  Fc | SOESRKS44VKRLhCD48Rev | CTTTAATCTTTTGACATCCCATTCTACAAT |
|  | SOESRKS44VKRLhCD48For | GTCAAAAGATTAAAGTACTTTGAATCCAAA |
| pCI-neo CD48 A2-  Fc | SOENST81ANIhCD48Rev | ACCCTCATAATGTAGATGTTGGCGTCCTCTTTCTGGAC |
|  | SOENST81ANIhCD48For | GTCCAGAAAGAGGACGCCAACATCTACATTATGAGGGT |
| pCI-neo CD48 A3-  Fc | SOEGN93MFhCD48Rev | TTCCATTCTTGCTCAAACATAGTCTTTTTCAACAC |
|  | SOEGN93MFhCD48For | GTGTTGAAAAAGACTATGTTTGAGCAAGAATGGAA |
| pCI-neo CD48 C4-  Fc | SOEE86DGN88MFhCD48Rev | TTGCTCAAACATAGTGTCTTTCAACACCCTC |
|  | SOEE86DGN88MFhCD48For | GAGGGTGTTGAAAGACACTATGTTTGAGCAA |
|  | SOEF34YhCD48For | CCTGGTTTTATACTTACGACCAGAAGATTGT |
|  | SOEF34YhCD48Rev | ACAATCTTCTGGTCGTAAGTATAAAACCAGG |
|  | SOEQ27EhCD48Rev | AAAACCAGGTTAGTTCTTTGTAGTTCTCAGG |
|  | SOEQ27EhCD48For | CCTGAGAACTACAAAGAACTAACCTGGTTTT |
|  | SOEK91EGN93MFhCD48Rev | TTCCATTCTTGCTCAAACATAGTCTCTTTCAACAC |
|  | SOEK91EGN93MF hCD48For | GTGTTGAAAGAGACTATGTTTGAGCAAGAATGGAA |
|  | SOENST81ANI hCD48Rev | ACCCTCATAATGTAGATGTTGGCGTCCTCTTTCTGGAC |
|  | SOENST81ANI hCD48For | GTCCAGAAAGAGGACGCCAACATCTACATTATGAGGGT |
|  | SOEK91E hCD48Rev | GCTCATTCCCAGTCTCTTTCAACACCCTCAT |
|  | SOEK91E hCD48For | ATGAGGGTGTTGAAAGAGACTGGGAATGAGC |
| pCI-neo CD48 C5-  Fc | SOESRKS44VKRLhCD48Rev | CTTTAATCTTTTGACATCCCATTCTACAAT |
|  | SOESRKS44VKRLhCD48For | GTCAAAAGATTAAAGTACTTTGAATCCAAA |
| pFUSE CD48-Fc | hCD48For | GAATTCATGTGCTCCAGAGGTTGGGAT |
|  | hCD48mFCRev | AGATCTTACTCCAAAGGACCGGGCCAG |
| pFUSE CD58-Fc | EcoRICD33sFor | GAATTCATGCCGCTGCTGCTACTGCTGCCC |
|  | SOEhCD58Rev | TGAGAAAGCTCTGAACTCAGAATTTTCCAG |
|  | SOEhCD58For | CTGGAAAATTCTGAGTTCAGAGCTTTCTCA |
|  | hCD58mFcRev | AGATCTTCTTGAATGACCGCTGCTTGG |
| pCI-neo hCD58-Fc | hCD58FcFor | GGATCCCTTTTCCCAACAAATATATGGT |
|  | hCD58FcRev | GGATCCACTTACCTGTTCTTGAATGACCGCTGCTTGG |
| pCI-neo hCD2-Fc | BamHIhCD2FcFor | GGATCCAAAAGAGATTACGAATGCCTTGGAA |
|  | BamHIhCD2FcRev | GGATCC ACTTACCTGT GTCCAGACCTTTCTCTGGACAGCT |

Supplementary table 1. Templates and primers employed in plasmid constructions by site directed mutagenesis.

| **Allele** | **Freq** |
| --- | --- |
| DRB1_0101 | 0.048581 |
| DRB1_0102 | 0.015284 |
| DRB1_0301 | 0.072938 |
| DRB1_0302 | 0.008567 |
| DRB1_0401 | 0.03004 |
| DRB1_0402 | 0.008593 |
| DRB1_0403 | 0.020916 |
| DRB1_0404 | 0.020473 |
| DRB1_0405 | 0.034716 |
| DRB1_0406 | 0.008291 |
| DRB1_0407 | 0.014421 |
| DRB1_0701 | 0.095725 |
| DRB1_0801 | 0.010942 |
| DRB1_0802 | 0.016259 |
| DRB1_0803 | 0.023349 |
| DRB1_0804 | 0.009089 |
| DRB1_0901 | 0.045288 |
| DRB1_1001 | 0.017347 |
| DRB1_1101 | 0.059725 |
| DRB1_1102 | 0.007167 |
| DRB1_1104 | 0.026471 |
| DRB1_1201 | 0.022978 |
| DRB1_1202 | 0.030382 |
| DRB1_1301 | 0.042651 |
| DRB1_1302 | 0.043978 |
| DRB1_1303 | 0.011286 |
| DRB1_1401 | 0.025283 |
| DRB1_1402 | 0.010527 |
| DRB1_1404 | 0.006084 |
| DRB1_1405 | 0.006058 |
| DRB1_1501 | 0.079008 |
| DRB1_1502 | 0.038644 |
| DRB1_1503 | 0.014835 |
| DRB1_1601 | 0.014789 |
| DRB1_1602 | 0.015587 |
| DRB3_0101 | 0.025 |
| DRB4_0101 | 0.025 |
| DRB5_0101 | 0.025 |
| HLA-DPA10103-DPB10201 | 0.025 |
| HLA-DPA10103-DPB10401 | 0.025 |
| HLA-DPA10201-DPB10101 | 0.025 |
| HLA-DPA10201-DPB10501 | 0.025 |
| HLA-DPA10301-DPB10402 | 0.025 |
| HLA-DQA10101-DQB10501 | 0.025 |
| HLA-DQA10102-DQB10602 | 0.025 |
| HLA-DQA10301-DQB10302 | 0.025 |
| HLA-DQA10401-DQB10402 | 0.025 |
| HLA-DQA10501-DQB10201 | 0.025 |
| HLA-DQA10501-DQB10301 | 0.025 |

Supplementary table 2. List of HLA alleles considered in the study and frequencies for the World population.
